# EIN2-dependent regulation of acetylation of histone H3K14 and non-canonical histone H3K23 in ethylene signalling

**DOI:** 10.1038/ncomms13018

**Published:** 2016-10-03

**Authors:** Fan Zhang, Bin Qi, Likai Wang, Bo Zhao, Siddharth Rode, Nathaniel D. Riggan, Joseph R. Ecker, Hong Qiao

**Affiliations:** 1 Department of Molecular Biosciences, Institute for Cellular and Molecular Biology, Austin, Texas 78712, USA; 2 Department of Molecular Biosciences, The University of Texas at Austin, Austin, Texas 78712, USA; 3 Plant Biology Laboratory, The Salk Institute for Biological Studies, La Jolla, California 92037 USA; 4 Howard Hughes Medical Institute, The Salk Institute for Biological Studies, La Jolla, California 92037 USA; 5 Department of Molecular Biosciences, The Center for Systems and Synthetic Biology, Austin, Texas 78712, USA

## Abstract

Ethylene gas is essential for many developmental processes and stress responses in plants. EIN2 plays a key role in ethylene signalling but its function remains enigmatic. Here, we show that ethylene specifically elevates acetylation of histone H3K14 and the non-canonical acetylation of H3K23 in etiolated seedlings. The up-regulation of these two histone marks positively correlates with ethylene-regulated transcription activation, and the elevation requires EIN2. Both EIN2 and EIN3 interact with a SANT domain protein named EIN2 nuclear associated protein 1 (ENAP1), overexpression of which results in elevation of histone acetylation and enhanced ethylene-inducible gene expression in an EIN2-dependent manner. On the basis of these findings we propose a model where, in the presence of ethylene, the EIN2 C terminus contributes to downstream signalling via the elevation of acetylation at H3K14 and H3K23. ENAP1 may potentially mediate ethylene-induced histone acetylation via its interactions with EIN2 C terminus.

## Abstract (web summary)

The translocation of the C-terminal domain of EIN2 to the nucleus is essential for induction of gene expression in response to the plant hormone ethylene. Here, Zhang *et al.* show that EIN2 is required for ethylene-inducible elevation of histone acetylation marks associated with transcriptional activation.

## Graphical abstract

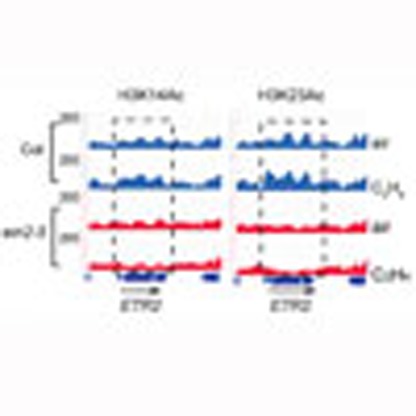

The plant hormone ethylene (C_2_H_4_) is essential for a myriad of physiological and developmental processes. It is important in the response to stresses such as drought, cold, flooding, pathogen infection^1^,^2^; and modulates stem cell division^1^. Interestingly, a recent study showed that the common aquatic ancestor of plants possessed the ethylene signalling pathway and shares similar functional mechanism as *Arabidopsis*^2^. Molecular genetic dissection has revealed that ethylene is perceived by a family of receptors bound to the membrane of the endoplasmic reticulum that are similar in sequence and structure to bacterial two-component histidine kinases^3^,^4^. Each receptor has an N-terminal transmembrane domain, and the receptors form dimers that bind ethylene via a copper cofactor, most likely provided by the copper transporter RESPONSIVE TO ANTAGONIST 1 (RAN1)^5^,^6^,^7^,^8^,^9^. Signalling from one of the receptors, ETHYLENE RESPONSE 1 (ETR1), is promoted by interaction with another ER-localized protein REVERSION-TO-ETHYLENE-SENSITIVITY 1 (RTE1)^10^. The ethylene receptors function redundantly to negatively regulate ethylene responses^11^ via a downstream Raf-like protein kinase called CONSTITUTIVE TRIPLE RESPONSE 1 (CTR1)^12^,^13^.

In the absence of ethylene, both the ethylene receptors and CTR1 are active, and CTR1 is associated with the ER membrane, where it directly interacts with ETR1 (refs 13, 14). Recent studies have shown that in the absence of ethylene, CTR1 phosphorylates ETHYLENE INSENSTIVE 2 (EIN2)^15^, which is an essential positive regulator of ethylene signalling. The CTR1-mediated phosphorylation of EIN2 C-terminal end (CEND) leads to the repression of EIN2 activity^15^. EIN2 has a large (∼800 amino acid) CEND, and its N-terminal region has a high degree of sequence identity with the NRAMP family of metal transporters^16^. In the absence of ethylene gas, EIN2 is localized to the ER membrane and interacts with ETR1 (ref. 17) and two F-box proteins called ETP1 and ETP2, which mediate protein degradation of EIN2 via the ubiquitin-proteasome pathway^18^. In the presence of ethylene gas, the CEND of EIN2 is dephosphorylated through an unknown mechanism. The dephosphorylated EIN2 CEND is cleaved and translocated into both the nucleus^15^,^19^,^20^ and the P-body. Interestingly, EIN2 C-terminal end mediates translation repression of EBF1 and EBF2 at P-body^21^,^22^. In the nucleus, the EIN2 CEND transduces signals to the transcription factors ETHYLENE INSENSTIVE 3 (EIN3) and ETHYLENE INSENSITIVE 3-LIKE 1 (EIL1), which are sufficient and necessary for activation of all ethylene-response genes^23^, leading to the ethylene-induced transcription activation.

Both genetics and molecular studies have demonstrated that EIN3 and EIL1 are the positive regulators of the ethylene response^23^,^24^,^25^. Most of the differential expressed genes in the presence of ethylene are up-regulated in an EIN3/EIL1-dependent manner^25^. EIN3 targets have been identified by chromatin immunoprecipitation, using a specific anti-EIN3 antibody, followed by high throughput sequencing (ChIP-seq)^25^, further confirming EIN3 is a positive regulator of ethylene signalling. Recent studies have provided significant insight into the molecular mechanisms involved in ethylene signaling^19^,^20^; however, the nuclear events after the EIN2 C-terminal end is translocated into the nucleus remain poorly understood.

In this study, we demonstrate that the acetylation of lysine 14 of histone H3 (H3K14Ac) and non-canonical acetylation of lysine 23 of histone H3 (H3K23Ac) are up-regulated by ethylene. Levels of these histone marks are positively associated with expression of a set of ethylene-regulated EIN3 targets as determined previously by ChIP-seq^25^. Most importantly, we demonstrated that EIN2 is required for the elevation of H3K14Ac and H3K23Ac. In addition, we found that the EIN2 CEND can interact with a SANT domain-containing protein we name EIN2 nuclear associated protein 1 (ENAP1). ENAP1 gain-of-function mutants display hyperacetylation of H3K14 and H3K23. Moreover, we found that EIN3 can interact with ENAP1. Although up-regulation of histone acetylation in response to ethylene is not EIN3/EIL1 dependent, transcription activation in the presence of ethylene is fully EIN3/EIL1 dependent. Overall, these findings provide evidence that increases in H3K14 acetylation and of the non-canonical H3K23 acetylation are positively associated with transcription activation in response to ethylene and that the elevation is EIN2 dependent.

## Results

### H3K14Ac and H3K23Ac levels are up-regulated by ethylene

**Figure 1:**
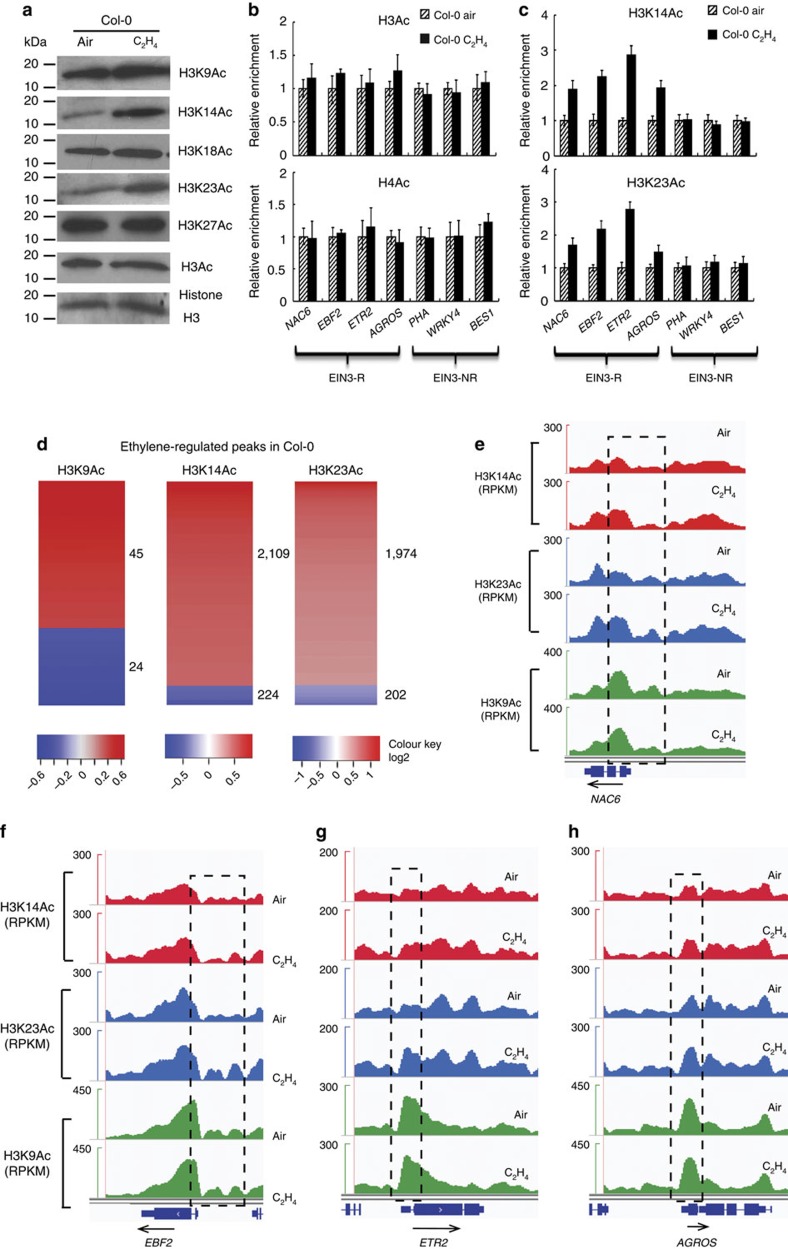
Acetylation at H3K14 and H3K23 is up-regulated by ethylene. (**a**) Global histone acetylation at indicated lysines of histone H3 in wild-type Col-0 seedlings grown in the dark for 3 days and then treated with or without 4 h ethylene gas. Total histones were subjected to immunoblotting with antibodies that recognize indicated histone H3 acetylations. Immunoblotting with anti-histone H3 was used as a loading control. (**b**–**c**) ChIP quantitative real time PCR detection of H3Ac, H4Ac, H3K14Ac and H3K23Ac in indicated EIN3-R genes and EIN3-NR genes^25^ in 3-day-old etiolated Col-0 seedlings with or without ethylene treatment. Precipitation with IgG pre-immune serum served as a control. Data represent relative fold changes. Each experiment has three biology replicates with similar result. (**d**) The levels of H3K9Ac (2 replications), H3K14Ac (1 replication) and H3K23Ac (2 replications) in Col-0 in response to ethylene examined by Standard ChIP-Seq assays (2 or 1 replication). Heat map showing ethylene-regulated peaks of H3K9Ac, H3K14Ac and H3K23Ac. (**e**–**h**) Standard ChIP-seq assays showing that the elevated enrichment of H3K14Ac and H3K23Ac in EIN3-R (4 representative genes indicated in the figure) in Col-0 treated with ethylene and no significant enrichment of H3K9Ac is observed. Binding levels are indicated by reads per kilobase per million reads in sample (RPKM). Col-0 seedlings grown in the dark for 3 days with or without 4 h ethylene gas treatment for ChIP-seq. Dash boxes highlight the difference enrichment of H3K9Ac, H3K14Ac or H3K23Ac. EIN3-R, represents ethylene-regulated EIN3 targets; EIN3-NR represents non-ethylene-regulated EIN3 targets. Dash boxes highlight the difference enrichment of H3K9Ac, H3K14Ac or H3K23Ac. Different letters were used to indicate statistically significance difference (*P*≦0.05 Student's *t*-test=3) within the same genes.

Transcriptional regulation is complicated and the status of chromatin clearly impacts levels of transcription. Histone acetylation is almost invariably associated with activation of transcription^26^, whereas deacetylation is associated with the repression of gene expression^27^. To investigate the connection between ethylene response and histone acetylation, we examined the global level of acetylated histone H3 by western blot in 3-day-old etiolated *Arabidopsis* Col-0 seedlings of treated with air or 4 h ethylene gas, and no significant differences were observed (Fig. 1a). In contrast to these global levels, the acetylation level of H3K14 and the non-canonical H3K23 (but not H3K9, H3K18 and H3K27) were significantly higher in the presence of ethylene gas than that of in the absence of ethylene gas (Fig. 1a).

To further study the connection between ethylene-triggered enrichment of H3K14Ac or H3K23Ac and transcription, we conducted chromatin immunoprecipitation coupled with quantitative PCR (ChIP-qPCR) using antibodies against H3Ac, H4Ac, H3K14Ac and H3K23Ac. This assay evaluated levels of histone acetylation in the promoter or 5′ UTR regions in those genes selected from two types of EIN3 targets: those not regulated by ethylene (EIN3-NR) and those up-regulated by ethylene (EIN3-R)^25^ (Supplementary Table 1). No significant enrichments of total acetylated histone H3 or total acetylated H4 were detected in promoter or 5′ UTR regions of genes in either category (Fig. 1b; Supplementary Fig. 1a). The acetylation at H3K14 and H3K23 were specifically enriched in the promoter or 5′ UTR regions of selected EIN3-R genes in the presence of ethylene gas (Fig. 1c), but the presence of ethylene did not alter levels of these marks at promoter or 5′ UTR regions of EIN3-NR genes (Supplementary Fig. 1a,b), indicating that acetylation of H3K14 and H3K23 may play some important roles in the activation of transcription in response to ethylene.

Our western blot result has shown globally increased histone acetylation H3K14Ac and H3K23Ac in response to ethylene, to further view the enrichment of H3K14Ac and H3K23Ac in response to ethylene gas in ethylene-regulated gene loci at genome-wide level, we performed ChIP-sequencing of H3K14Ac, H3K23Ac and H3K9Ac using chromatin isolated from 3-day-old etiolated Col-0 seedlings treated with or without 10 parts per million (p.p.m.) ethylene gas for 4 h and analyzed used pooled reads (see ‘Methods' section). In our analysis, similar differential peak numbers were called for each histone mark in response to ethylene (Supplementary Tables 2, 3 and Supplementary Data 1a–j). For the H3K9Ac mark, only 69 different DNA regions (peaks) (|M|>=0.4 and FDR<0.2) were associated with significant differences in levels between air and ethylene treatments (Fig. 1d; Supplementary Data 2a). In contrast, for H3K23Ac and H3K14Ac, 2176 and 2333 peaks showed differential enrichment after ethylene treatment (|M|>=0.4 and FDR<0.2), and at the majority of sites, acetylation was up-regulated by ethylene treatment (Fig. 1d; Supplementary Table 3; Supplementary Data 2b–c). Gene ontology (GO) analysis of genes associated with up-regulated peaks of H3K14 Ac and H3K23Ac (Supplementary Data 2e,f) showed enrichment for the GO term ‘response to ethylene stimulus' (Supplementary Fig. 1c,d; Supplementary Data 2e,f). But there was no enrichment for a particular GO term in the set of genes, which associated with up-regulated H3K9Ac peaks by ethylene treatment (Supplementary Fig. 1e; Supplementary Data 2d).

The ethylene-regulated enrichment of H3K14Ac and H3K23Ac in EIN3-R genes, and not in EIN3-NR genes, was also confirmed genome-wide. As shown in Fig. 1e–h, the ethylene-induced enrichment of H3K14Ac and H3K23Ac occurred in EIN3-R genes. However no enrichment was observed in EIN3-NR (Supplementary Fig. 1f–h). The elevation of H3K14Ac and H3K23Ac were detected in about 60% (133/224) of EIN3 targets that were up-regulated by ethylene^25^ (Supplementary Fig. 1i). Collectively, these results support the conclusion that ethylene specifically elevates H3K14Ac and non-canonical H3K23Ac in a subset of the EIN3 targeted genes that are regulated at the transcriptional level by ethylene.

### Ethylene-induced H3K14 and H3K23 acetylation requires EIN2

**Figure 2:**
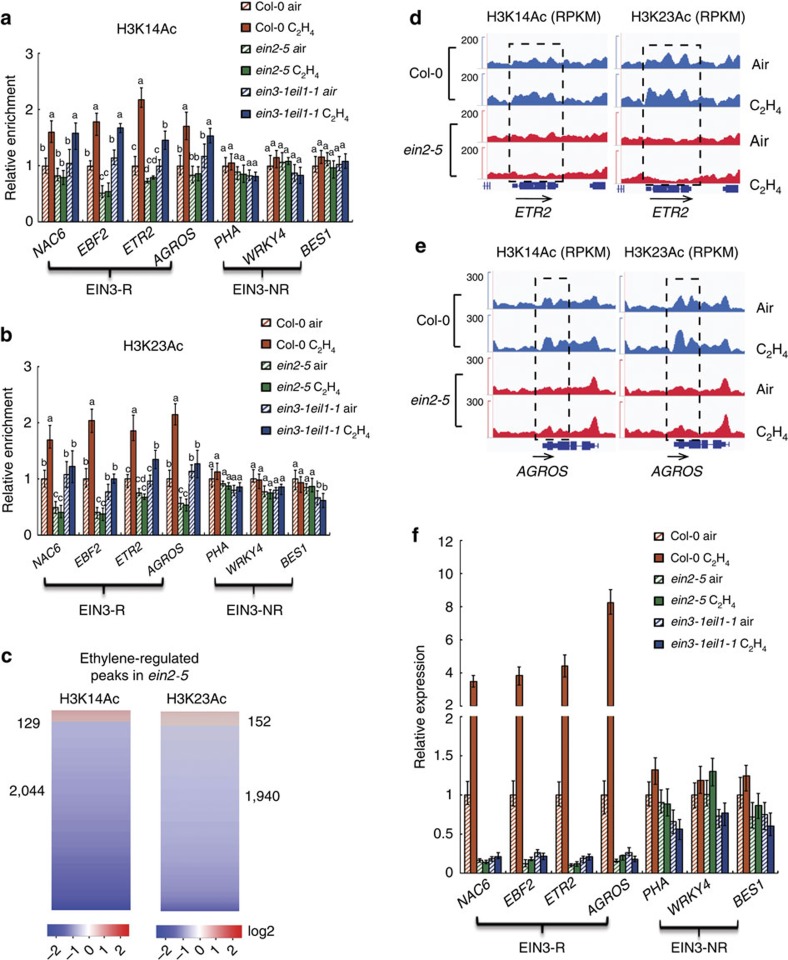
EIN2 is important for the elevation of H3K14Ac and H3K23Ac in the presence of ethylene. (**a**–**b**) ChIP quantitative real time PCR detection of H3K14Ac and H3K23Ac enrichment in Col-0, *ein2-5, ein3-1eil1-1* 3-day-old etiolated seedlings treated with air or 4 h ethylene gas. Precipitation with IgG pre-immune serum served as a control. Data represent the relative fold change. EIN3-R, represents ethylene-regulated EIN3 targets; EIN3-NR represents non-ethylene-regulated EIN3 targets. Each experiment has three biology replicates with similar result. (**c**) The levels of H3K14Ac (2 replications) and H3K23Ac (2 replications) in *ein2-5* in response to ethylene examined by Standard ChIP-seq assays. Heat map showing ethylene-regulated peaks of H3K14Ac and H3K23Ac. (**d**,**e**) Standard ChIP-seq assays showing that ethylene- induced enrichment of H3K14Ac and H3K23Ac in EIN3-R genes is abolished in *ein2-5* mutant. Binding levels are indicated by reads per kilobase per million reads in sample (RPKM). Col-0 seedlings grown in the dark for 3 days with or without 4 h ethylene gas treatment for Chip-seq. Dash boxes highlight the difference enrichment of H3K9Ac, H3K14Ac or H3K23Ac. (**f**) The expression of ethylene-regulated genes is positively associated with the enrichment of H3K14Ac and H3K23Ac. Total RNAs were extracted from 3-day-old etiolated seedlings from indicated genotypes and gene expression was analyzed by qualitative RT-PCR (3 biological replicates). EIN3-R, represents ethylene-regulated EIN3 targets; EIN3-NR represents non-ethylene-regulated EIN3 targets. Different letters were used to indicate statistically significance difference (*P*≦0.05 Student's *t*-test=3) within the same genes.

To evaluate the role of EIN2 in the observed elevations of H3K14Ac and H3K23Ac in ethylene-regulated genes, we examined histone acetylation in the promoters of EIN3-R genes and EIN3-NR genes in *ein2-5* and *ein3-1eil1-1* mutants by ChIP-qPCR after treatment with air or ethylene gas for 4 h. The levels of H3K14Ac and H3K23Ac in the promoter or 5′ UTR regions of the EIN3-R genes examined, but not in the promoter or 5′ UTR regions of EIN3-NR genes, were decreased in *ein2-5* mutant seedlings even without ethylene treatment (Fig. 2a,b; Supplementary Fig. 2a). In addition, the ethylene-induced elevation of H3K14Ac and H3K23Ac in EIN3-R genes was completely impaired in *ein2-5* mutant (Fig. 2a,b; Supplementary Fig. 2a). Interestingly, in *ein3-1eil1-1* mutant seedlings, the elevation of H3K14Ac and H3K23Ac in the promoter or 5′ UTR regions of EIN3-R genes appeared to be slightly impaired though this was variable between genes. However, the basal levels were comparable to that in Col-0 seedlings (Fig. 2a,b; Supplementary Fig. 2a). These results indicate that ethylene can induce H3K14 and non-canonical H3K23 acetylation and it requires EIN2.

To further confirm the role of EIN2 CEND in ethylene-regulated elevation of H3K14Ac and H3K23Ac at the genome-wide level, we conducted ChIP-seq to analyze H3K14Ac, H3K23Ac levels in chromatin isolated from 3-day-old etiolated *ein2-5* mutant seedlings treated with ethylene or air. Notably, in this mutant, most of the differential peaks of H3K14Ac and H3K23Ac were decreased in the presence of ethylene (Fig. 2c; Supplementary Data 3a,b). The ethylene-induced enrichment of H3K14Ac and H3K23Ac observed in EIN3-R genes in the wild-type seedlings was impaired in the *ein2-5* mutant, and the basal levels of these marks were lower than those in Col-0 seedlings (Fig. 2d,e; Supplementary Fig. 2b,c). No significant differences in H3K14Ac and H3K23Ac levels were observed in the EIN3-NR subset of genes (Supplementary Fig. 2d–f). We then conducted ChIP-qPCR assays of H3K14Ac and H3K23Ac in the *EIN2* gain-of-function mutant (*EIN2ox*). Both marks are enriched in EIN3-R gene without ethylene treatment. However, more enrichment was detected in the presence ethylene gas. But, the level of both marks in EIN3-NR genes was not significantly altered (Supplementary Fig. 2g), further suggesting that EIN2 is required for the regulation of acetylation of H3K14 and H3K23 in response to ethylene.

We next conducted quantitative RT-PCR to examine the gene expression of both EIN3-R and EIN3-NR in Col-0, *ein2-5* and *ein3-1eil1-1* seedlings treated with or without ethylene gas for 4 h. In Col-0 and *ein2-5* plants, gene expression was positively associated with the level of H3K14Ac and H3K23Ac in response to ethylene (Fig. 2a,b,d,e). However, in the *ein3-1eil1-1* mutant, induction of gene expression by ethylene was completely abolished, even though the enrichment of H3K14Ac and H3K23Ac was detectable in the presence of ethylene (Fig. 2f). This is in agreement with previous studies that EIN3 and EIL1 play an important role in transcriptional regulation^24^,^25^. Taken together, our data demonstrate that the elevation of H3K14Ac and H3K23Ac at EIN3-R is ethylene specific and the levels of these histone modifications are positively correlated with gene activation in response to ethylene. EIN2 is appears to be required to the maintain basal levels of histone acetylation and for ethylene-induced elevation of histone acetylation.

### A SANT domain-containing protein interacts EIN2 CEND

**Figure 3:**
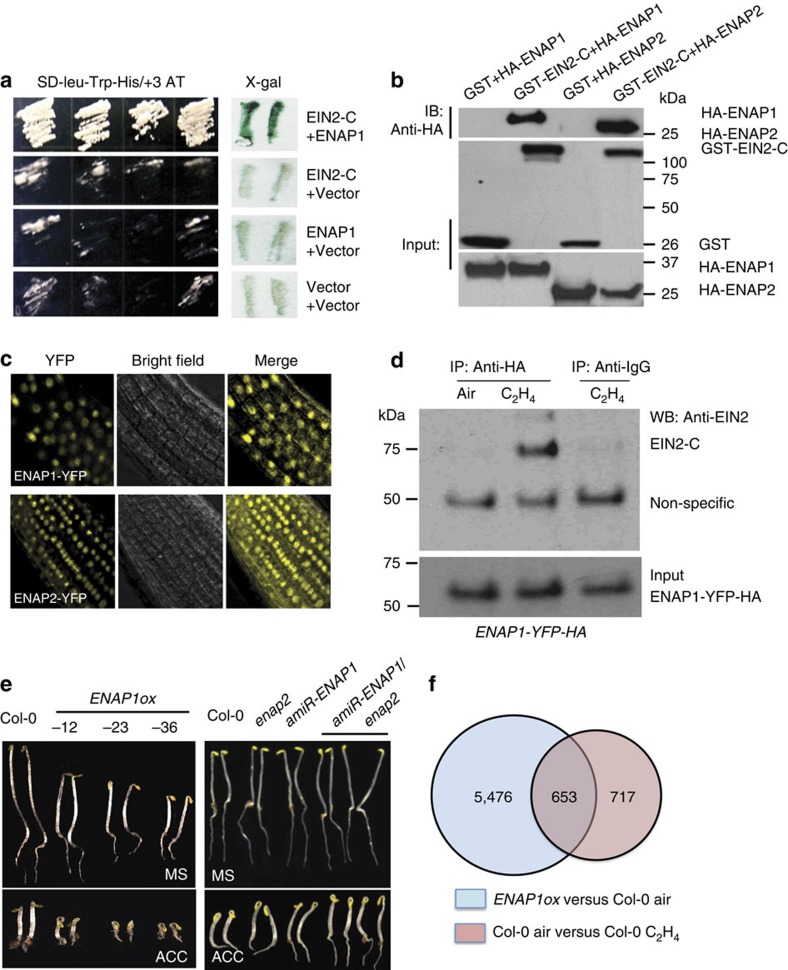
EIN2 C-terminal end interacts with ENAP1 and ENAP1 is involved in ethylene response. (**a**) Yeast two-hybrid assay revealed an interaction between the EIN2 C-terminal end and ENAP1. Growth on selective plates lacking leucine, histidine, tryptophan with 20 mM 3-AT (−Leu, −Trp, −His, +3-AT) (left panel) and an X-Gal filter assay was performed to verify the interaction between EIN2 CEND and ENAP1 (right panel). (**b**) *In vitro* GST pull-down showing the interaction between EIN2 CEND and ENAP1 and ENAP2. (**c**) Both ENAP1 and ENAP2 are localized to the nucleus. The images were taken from the roots of 3-day-old etiolated seedlings of *pENAP1*:*ENAP1-YFP* and *pENAP2*:*ENAP2-YFP* under a confocal microscope. (**d**) ENAP1 interacts with EIN2 CEND *in vivo* in the presence of ethylene gas. Total protein extracts from 35 S:*ENAP1-YFP-HA* transgenic plants treated with or without ethylene were immunoprecipitated with an anti-HA antibody. The EIN2 CEND was detected by anti-EIN2 CEND antibody. The immunoprecipitation with IgG from 35S:*ENAP1-YFP-HA* transgenic plants treated with ethylene was used as a control. (**e**) Phenotype of *ENAP1* gain-of-function (*ENAp1ox*) and ENAP1-deficient *amiR-ENAP1/enap2* mutants. The plants indicated in the figure were grown 3 days on MS with or without 10 μM ACC before being photographed. (**f**) *ENAP1ox* plants show transcriptional activation of ethylene-response genes. Total RNA was prepared from 3-day-old etiolated seedlings of *ENAP1ox* or wild-type (Col-0) plants treated with air or 4 h ethylene. Differentially expressed genes were identified by fragments per kilobase per million reads (FPKM) filter<0.1, requiring a twofold change comparing the indicated conditions with *P*<=0.05 after Benjamini–Hochberg correction.

The EIN2 CEND does not have known functional motifs. In an effort to clarify the function of this domain, we conducted yeast two-hybrid screening using the EIN2 CEND (from 808 aa through 1294 aa of EIN2). A novel protein encoded by *at3g11100* was isolated from this screen (Fig. 3a; Supplementary Fig. 3A). As the protein is predicted to localize in the nucleus, we named it EIN2 nuclear associated protein 1 (ENAP1). Through a motif search, a SANT domain was identified in ENAP1 (Supplementary Fig. 3a). The SANT domain is conserved from yeast to human in many chromatin remodelling enzymes such as SWI3, N-COR, and GCN5 (refs 28, 29). This domain is known to interact with histone tails and is important for chromatin remodelling.

Sequence analysis revealed the presence of a paralogous gene, named as *ENAP2* (*at5g05550*), which is 72% identical to ENAP1 at the amino acid sequence level (Supplementary Fig. 3A). glutathione S-transferase (GST) pull-down assays showed that both ENAP1 and ENAP2 interact with EIN2 CEND *in vitro* (Fig. 3b). We first examined the cellular localization of ENAP1 and ENAP2 by expressing the proteins fused with a YFP tag from the native promoter (*pENAP1*:*ENAP1-YFP*; *pENAP2*:*ENAP2-YFP*) in Col-0. As we expected, both ENAP1 and ENAP2 localized exclusively to the nucleus (Fig. 3c). To further confirm the interaction between EIN2 C-terminal end and ENAP1 *in vivo*, we performed an immunoprecipitation assay in YFP-HA-tagged ENAP1 (*ENAP1-YFP-HA*) transgenic plants. As shown in Fig. 3e, the interaction between EIN2 CEND and ENAP1 was detected only in the presence of ethylene gas, suggesting that the interaction occurs in the nucleus.

To examine the function of the ENAPs in the ethylene response genetically, we first generated gain-of-function mutants of *ENAP1* (*ENAP1ox*) and *ENAP2* (*ENAP2ox*) by introducing *ENAP1* or *ENAP2* driven by CaMV35S promoter into the Col-0 background. Interestingly, *ENAP1ox* seedlings displayed shorter, thicker hypocotyls and some lines had more pronounced apical hooks (Fig. 3e). These phenotypes are consistent with an enhanced response to ethylene even in the absence of exogenous ethylene treatment. In the presence of ethylene, the 3-day-old etiolated seedlings displayed shorter, thicker hypocotyls and some lines had more pronounced apical hooks compared with Col-0 plants (Fig. 3e). However, no obvious ethylene-responsive phenotype was observed for *ENAP2ox* (Supplementary Fig. 3c). The phenotypes of *EAP1ox* are consistent with an enhanced ethylene-response phenotype, however, it is possible that *ENAP1ox* also affects processes that are not directly related to the ethylene response and that may also contribute to the phenotype. We used an artificial microRNA approach previously described^18^,^30^ to specifically inhibit expression of *ENAP1* (*amiR-ENAP1*). As shown in Supplementary Fig. 3d, inhibition of *ENAP1* expression resulted in a phenotype consistent with reduced ethylene response, while the expression of *ENAP2* was not affected (Supplementary Fig. 3b). We then further crossed *amiR-ENAP1* into *enap2* mutant and obtained *amiR-ENAP1/enap2,* which showing significant reduced ethylene sensitivity compared with Col-0 (Fig. 3e). Thus, two independent genetic approaches suggest that ENAP1 may be involved in the ethylene response. To further examine the function of ENAP1 at the molecular level, genome-wide gene expression was examined in *ENAP1ox* without ethylene treatment. Transcriptome analysis revealed that more than 40% of genes differentially expressed in wild-type, ethylene-treated plants overlapped with genes differentially expressed in *ENAP1ox* plants (*P*<1 × 10^–200^, using Fisher's exact test), providing additional evidence that ENAP1 positively regulates the ethylene response (Fig. 3f; Supplementary Data 4a,b).

### ENAP1 interacts with histone H3 and enhances acetylation

**Figure 4:**
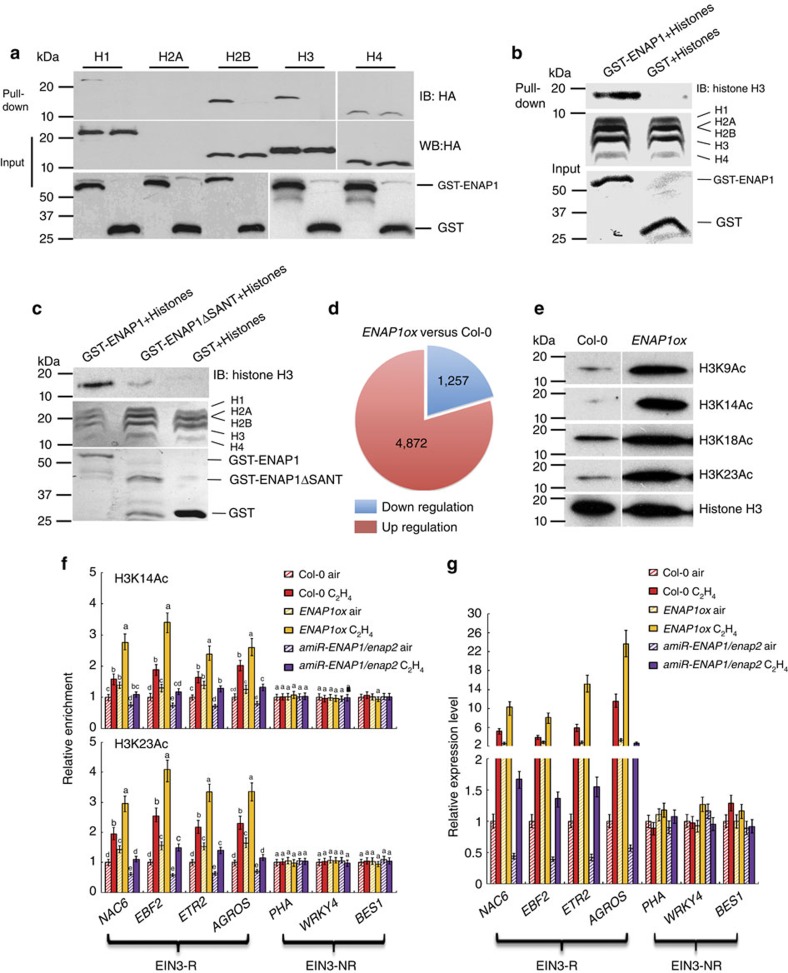
ENAP1 interacts with histone H3 and regulates histone acetylation of H3K14 and H3K23. (**a**) *In vitro* GST pull-down assay showing that ENAP1 interacts with histone H3 and H2B. (**b**) GST-labelled ENAP1 interacts with histone H3 in nucleosomes isolated from cow cell extracts. Purified GST-ENAP1 from *E. coli* was incubated with calf thymus nucleosomes, the pull-down products were subjected to immunoblotting with anti-histone H3 antibody. (**c**) The SANT domain is required for the interaction between ENAP1 and histone H3. GST-labelled full-length ENAP1 or truncated ENAP1ΔSANT expressed from *E. coli* was used for GST pull-down. The pull-down products were subjected to immunoblotting with anti-histone H3 antibody. (**d**) RNA-seq data collected from Fig. 3g in *ENAP1ox* or wild-type (Col-0) plants treated with air or 4 h ethylene was analyzed for the up- and down-regulated gene numbers. (**e**) Total histone extractions from 3-day-old etiolated seedlings of Col-0 and *ENAP1ox* seedlings were subjected to the immunoblotting with antibodies indicated. Immunoblotting with anti-histone H3 antibody served as a loading control. (**f**) ChIP-qPCR to quantify H3K14Ac and H3K23Ac enrichment in Col-0, *ENAP1ox* and *amiR-ENAP1/enap2* plants treated with air or 4 h ethylene gas in indicated EIN3-R genes and EIN3-NR genes. Precipitation with IgG pre-immune serum served as a control. Data represent the relative fold change. Each experiment has three biology replicates with similar result. (**g**). Quantitative PCR detection of EIN3 target gene expression in Col-0, *ENAP1ox* and *amiR-ENAP1/enap2* plants treated with air or 4 h ethylene gas (three biological replicates). EIN3-R, represents ethylene-regulated EIN3 targets; EIN3-NR represents non-ethylene-regulated EIN3 targets. Different letters were used to indicate statistically significance difference (*P*≦0.05 student's *t*-test=3) within the same genes.

Because of the presence of the SANT domain, which mediates the interactions of a number of proteins with histone tails^28^,^29^, we tested for an interaction between ENAP1 and histones. The coding sequences of genes for histones H1 (ref. 31), H2A^32^,^33^ H2B^34^, H3 (ref. 35) and H4 (ref. 36) and ENAP1 were cloned into wheat germ expression vectors and expressed in a wheat germ protein expression system. Other than H2A, all the other histone proteins are well expressed, and a GST pull-down assay was used to evaluate interactions. As shown in Fig. 4a and Supplementary Fig. 4b, ENAP1 interacted with naked histone H3, and weakly interacted with naked histone H2B *in vitro*. When GST-ENAP1 was incubated with histones core from calf thymus, ENAP1 interacted with histone H3 in the nucleosome as well (Fig. 4b).

To examine the roles of the SANT domain in binding histone H3, *in vitro* GST pull-down was conducted using full length of ENAP1 and a truncated ENAP1, which does not contain the SANT domain (ENAP1ΔSANT) (Supplementary Fig. 4a). In the absence of the SANT domain, the interaction between ENAP1 with histone H3 was barely detectable (Fig. 4c). We then conducted transcriptome analysis of *ENAP1ox* plants (Fig. 3), of the approximately 6,000 genes differentially regulated in *ENAP1ox* compared with Col-0 plants, about 80% were up-regulated (Fig. 4d), suggesting that ENAP1 positively regulates gene transcription.

In general, histone acetylation is a gene activation marker, and we speculate that the positive regulation of transcription in *ENAP1ox* seedlings is due to the increase in histone acetylation. To test this hypothesis, we examined the global level of histone acetylation (H3K9Ac, H3K14Ac, H3K18Ac, H3K23Ac) in *ENAP1ox* plants. We found that each of the histone acetylations examined was elevated in the ENAP1 gain-of-function mutant (Fig. 4e). This suggests that ENAP1 is involved in the regulation of histone acetylation (H3K9AC, K14Ac, K18Ac and K23Ac) in general, raising the question of how ENAP1 functions in the ethylene response.

We next examined the acetylation of H3K14 and H3K23 in the promoter or 5′ UTR regions of EIN3-R genes and EIN3-NR genes in Col-0 and in *ENAP1* gain-of-function and knocking down line*s* by ChIP-qPCR. In the absence of ethylene gas, the levels of H3K14Ac and H3H23Ac were slightly elevated relative to those in wild-type plants in *ENAP1ox* plants and were decreased in *amiR-ENAP1* plants in the promoter or 5′ UTR regions of EIN3-R genes; there were no differences in promoter or 5′ UTR regions of EIN3-NR genes (Fig. 4f; Supplementary Fig. 4c). In the presence of ethylene gas, the levels of H3K14Ac and H3K23Ac in the gain-of-function *ENAP1ox* mutant plants were greater than those in Col-0 plants. But in *amiR-ENAP1* plants, the levels of H3K14Ac and H3K23Ac were lower than those in Col-0 plants, although the levels were still slightly elevated by ethylene gas (Fig. 4f; Supplementary Fig. 4b).

To further examine the association of the H3K14Ac and H3K23Ac histone marks and gene transcription activation in ethylene signalling, total RNAs from *ENAP1ox* and *enap1* mutant plants treated with or without ethylene gas were analyzed by quantitative RT-PCR for expression of EIN3-R and EIN3-NR genes. The expression of EIN3-R genes was positively associated with the levels of H3K14Ac and H3K23Ac and was enhanced by ethylene treatment (Fig. 4f,g). The expression of EIN3-NR genes did not differ significantly. Taken together, our data suggest that ENAP1 enhances histone H3 acetylation at K14 and K23 in response to ethylene which is positively correlated with expression of ethylene-responsive genes.

### *ein2-5* and *ein3-1eil1-1* partially rescue the *ENAP1ox* phenotype

**Figure 5:**
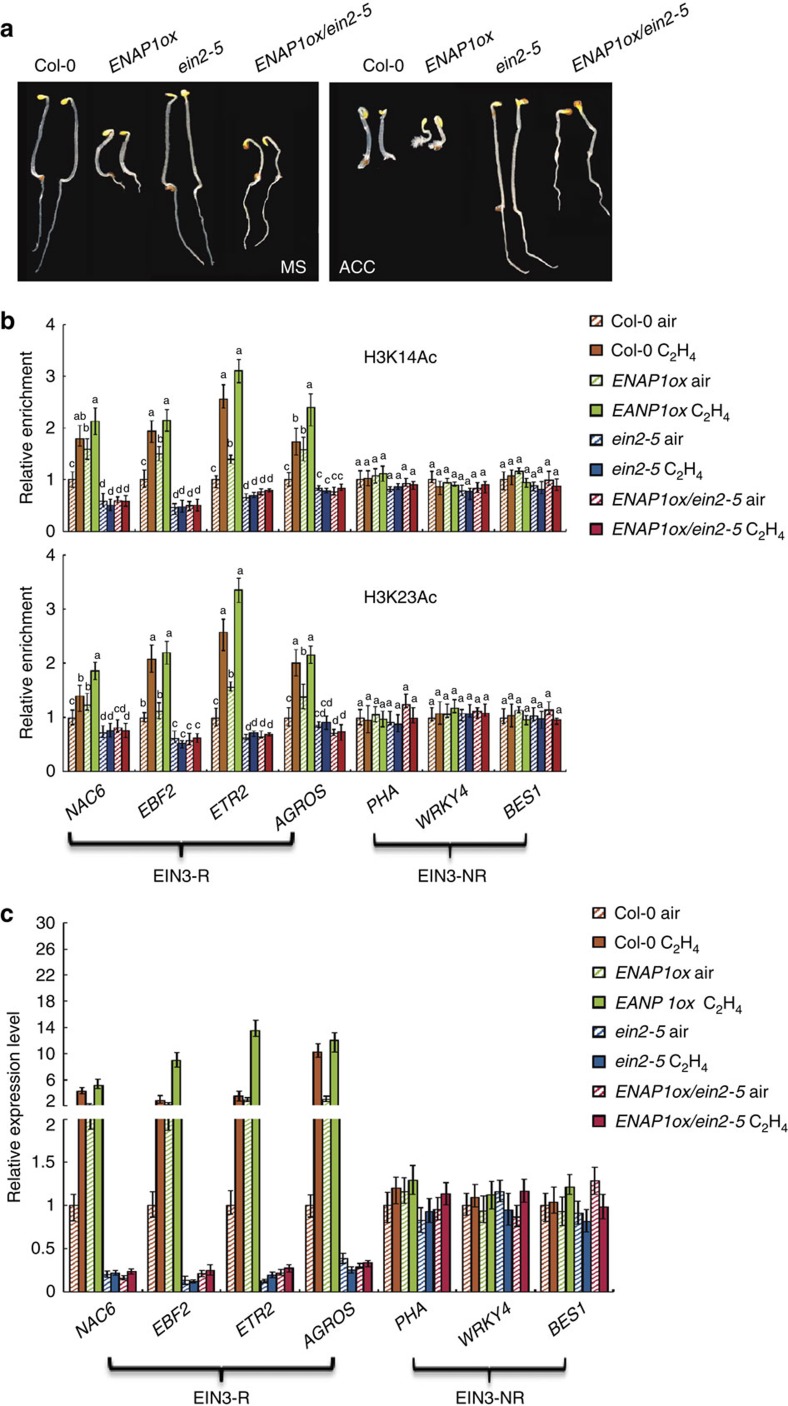
Partial rescue of the *ENAP1ox* phenotype by *ein2-5*. (**a**) The phenotype of *ENAP1ox* in the *ein2-5* mutant background. The 3-day-old etiolated seedlings were grown on MS with (right panel) or without (left panel) 10 μM ACC before being photographed. (**b**) ChIP-qPCR to quantify H3K14Ac and H3K23Ac enrichment in Col-0, *ENAP1ox*, *ein2-5* and *ENAP1ox/ein2-5* plants treated with air or 4 h ethylene gas in indicated EIN3-R genes and EIN3-NR genes. Precipitation with IgG pre-immune serum served as a control. Data represent the relative fold change. Each experiment has three biology replicates with similar result. (**c**) Quantitative PCR detection of EIN3 target gene expression in Col-0, *ENAP1ox*, and *ENAP1ox/ein2-5* seedlings grown in air or 4 h ethylene gas (three biological replicates). EIN3-R, represents ethylene-regulated EIN3 targets; EIN3-NR represents non-ethylene-regulated EIN3 targets. Different letters were used to indicate statistically significance difference (*P*≦0.05, Student's *t*-test=3) within the same genes.

We hypothesized that ENAP1 functions in ethylene signalling through an interaction with EIN2. To test our hypothesis, *ENAP1ox* was crossed into the *ein2-5* mutant (*ENAP1ox/ein2-5*). In the presence of ethylene, *ENAP1ox/ein2-5* had longer roots and less pronounced apical hooks compared with *ENAP1ox* (Fig. 5a). Hypocotyl length was increased though still shorter than *ein2-5*. This is consistent with a partial rescue of the phenotype of *ENAP1ox* by *ein2-5*. The *ENAP1ox/ein2-5* seedlings had similar phenotypes in the presence ethylene as in the absence of ethylene with no obvious apical hook, elongated roots and partial recovery of hypocotyl length compared with that of *ENAP1ox* (Fig. 5a). Levels of ENAP1 protein were not affected by the deficiency in EIN2 or by ethylene (Supplementary Fig. 5a). We then examined the levels of H3K14Ac and H3K23Ac in EIN3-R and EIN3-NR from *ENAP1ox* and *ENAP1ox/ein2-5* mutants treated with air or ethylene for 4 h. In *ENAP1ox/ein2-5* seedlings, no enhancement of H3K14Ac and H3K23Ac in EIN3-R genes with the presence of either air or 4 h ethylene gas was observed as when *ENAP1* was over-expressed. Whereas the levels of both histone marks in EIN3-R were similar in *ENAP1ox/ein2-5* as in the *ein2-5* mutant. In addition, the level of both marks in EIN3-NR genes was not significantly altered (Fig. 5b; Supplementary Fig. 5b). The enhanced elevation of EIN3-R gene expression caused by *ENAP1ox* was also abolished in *ENAP1ox/ein2-5* regardless of the presence of 4 h ethylene gas, whereas the expression of EIN3-NR genes was not significantly altered in mutants relative to wild-type seedlings (Fig. 5c). In addition, to validate whether the ethylene biosynthetic pathway is involved in the phenotype of *ENAP1* overexpression, we examined the altered genes of *ENAP1ox* in the presence ethylene biosynthetic inhibitor aminoethoxyvinylglycine (AVG). No significant change was detected in the presence of AVG (Supplementary Fig. 5c). Thus, both our genetic and molecular data suggest that at least part of the *ENAP1ox* morphological, enhanced histone acetylation and ethylene-inducible gene expression phenotypes depend on EIN2.

**Figure 6:**
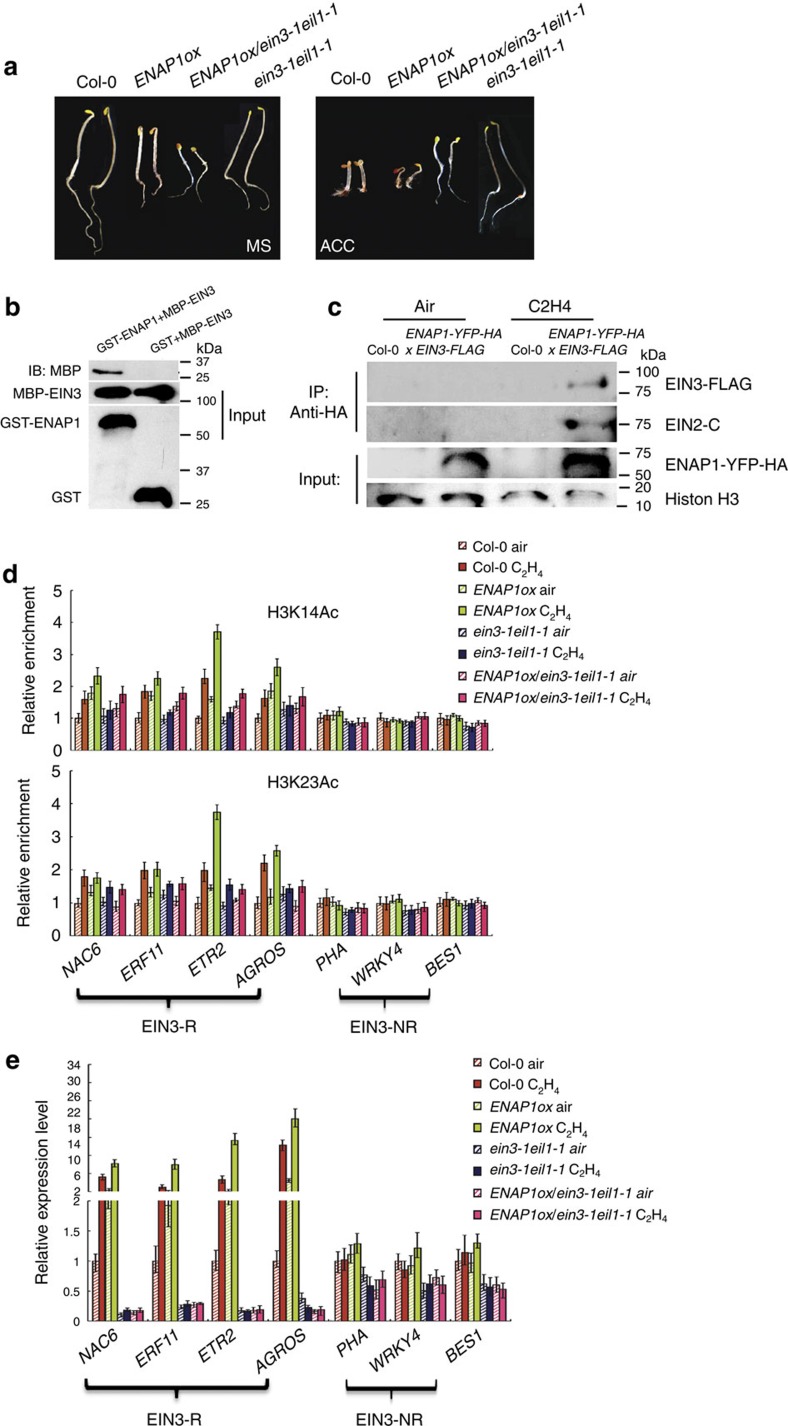
Partial rescue of the *ENAP1ox* phenotype by *ein3-1eil1-1*. (**a**) The phenotype of *ENAP1ox/ein3-1eil1-1* mutant. The 3-day-old etiolated seedlings of the plants indicated in the figure were grown on MS with (right panel) or without (left panel) 10 μM ACC before photographed. (**b**) *In vitro* GST pull-down showing the interaction between EIN3 and ENAP1. GST-tagged ENAP1 and MBP-tagged EIN3 purified from *E. coli* were used. (**c**) ENAP1 interacts with EIN3 *in vivo* in the presence of ethylene. Total protein extracts from 35S:*ENAP1-YFP-HA*/*pEIN3*:*EIN3-FLAG* transgenic plants treated with or without ethylene treatment were immunoprecipitated with an anti-HA antibody. EIN2 was detected with an anti-EIN2 CEND antibody, and EIN3 was detected using an anti-FLAG antibody. (**d**) ChIP-qPCR to quantify H3K14Ac and H3K23Ac enrichment in Col-0, *ENAP1ox*, *ein3-1eil1-1* and *ENAP1ox/ein3-1eil1-1* plants treated with air or 4 h ethylene gas in indicated EIN3-R genes and EIN3-NR genes. Precipitation with IgG pre-immune serum served as a control. Data represent the relative fold change. Each experiment has three biological replicates which showed similar results. (**e**) Quantitative PCR detection of EIN3 target gene expression in Col-0, *ENAP1ox*, *ein3-1eil1-1* and *ENAP1ox/ein3-1eil1-1* seedlings grown in air or 4 h ethylene gas (three biological replicates). EIN3-R, represents ethylene-regulated EIN3 targets; EIN3-NR represents non-ethylene-regulated EIN3 targets. Different letters were used to indicate statistically significance difference (*P*≦0.05 Student's *t*-test=3) within the same genes.

Transcription initiation is a stepwise process requires many proteins. In general, histone modifications make DNA more or less accessible to the transcription factors that control gene expression. To examine the connection between ENAP1 and EIN3/EIL1, *ENAP1ox* was crossed with *ein3-1eil1-1* to obtain the *ENAP1ox/ein3-1eil1-1* mutant. Interestingly, *ENAP1ox/ein3-1eil1-1* displayed a phenotype similar to that of *ENAP1ox/ein2-5* with and without ethylene (Fig. 6a; Supplementary Fig. 6a,b). Moreover, the ENAP1 protein level was similar in *ENAP1ox/ein3-1eil1-1* as in wild-type seedlings (Supplementary Fig. 5a), showing that EIN3/EIL1 is not required for the protein level of ENAP1, but may contribute to the *ENAP1ox* phenotype. The physical interaction between ENAP1 and EIN3 were examined by both *in vitro* GST pull-down and *in vivo* immunoprecipitation. ENAP1 interacts with EIN3 both *in vitro* and *in vivo* in the presence of ethylene (Fig. 6b,c), suggesting that EIN2, ENAP1 and EIN3 may function in the same complex.

To further study how EIN3 may regulate ethylene-induced acetylation at H3K14 and H3K23, ChIP-qPCR was performed to study the presence of these modifications on EIN3-R and EIN3-NR genes in *ein3-1eil1-1* and *ENAP1ox*/*ein3-1eil1-1* seedlings treated with air or ethylene gas for 4 h. As shown in Fig. 2d, H3K14Ac and H3K23Ac levels were elevated on at least some EIN3-R genes in *ein3-1eil1-1* although there was some variability between experiments. However, compared with the levels of H3K14Ac and H3K23Ac at EIN3-R genes in *ENAP1ox* in the presence of ethylene, the levels in *ENAP1ox*/*ein3-1eil1-1* are significantly decreased (Fig. 6d; Supplementary Fig. 6c), suggesting that EIN3 may contribute to the ethylene-induced enhancement of H3K14 and H3K23 acetylation seen in *ENAP1ox*.

When transcripts from EIN3-R and EIN3-NR genes in *ENAP1ox*/*ein3-1eil1-1* seedlings were quantified, we found that ethylene-induced expression of EIN3-R genes was completely abolished in the plants treated with or without 4 h ethylene gas (Fig. 6e). Interestingly, levels of H3K14Ac and H3K23Ac in these genes in *ENAP1ox/ein3-1ei1-1* seedlings were comparable to those in Col-0 seedlings (Fig. 6d), confirming that EIN3 and EIL1 are essential for transcriptional activation during ethylene signalling as previously reported^23^,^37^,^38^,^39^.

## Discussion

Ethylene gas is essential for developmental processes and stress responses in plants. Previous studies have demonstrated that the transcription factors EIN3 and EIL1 are necessary for activation of gene expression in response to ethylene^23^. The C-terminal END of EIN2 is translocated into the nucleus to trigger EIN3/EIL1-dependent transcription activation^15^,^19^,^20^. Exactly how EIN2 CEND functions in the nucleus is still unknown. The isolation of EER3 or PHB3, which is an enhancer of ethylene response and interacts with EIN2 CEND, indicating that RNA splicing may be involved in ethylene response^40^. In addition, another ethylene-response enhancer, EER5, proteasome COP9 initiation factor (PCI/PINT)-associated module, interacts with EIN2 CEND, may function in modification or degradation of target proteins^41^. In this study, we propose that EIN2 CEND may be guided to specific gene targets where it interacts with ENAP1 to elevate the level of histone H3 acetylation at K14 and the non-canonical K23, initiating EIN3-dependent activation of transcription. Importantly, only the acetylation at K14 and non-canonical K23 are differentially regulated by ethylene among the five lysine residues of histone H3 examined (H3K9, H3K14, H3K23, H3K18, H3K27). A recent study showed that the absence of canonical histone marks is not likely related to inducible gene expression^42^; however, non-canonical histone marks were not explored. Our study provides evidence that acetylation at specific lysine residues is regulated by ethylene and that the elevation is associated with ethylene-induced transcriptional activation (Figs 1 and 2).

Our genetic and molecular analyses demonstrated that EIN2 is required, at least at a subset of loci, for the regulation of the elevation of H3K14Ac and H3K23Ac induced by ethylene (Figs 2, 5; Supplementary Figs 2, 5). Interestingly, in the absence of EIN2, the level of H3K14Ac and H3K23Ac at many loci is reduced, one possibility is that EIN2 is important for specificity and once EIN2 is removed, specificity is impaired and therefore H3K14Ac and H3K23Ac are reduced at many loci. Alternatively, it could due to a large-scale chromatin structural change caused by loss of function of EIN2. Further study on the function of EIN2 will provide more insight. EIN2 does not contain any known histone recognition motifs. We hypothesize that EIN2 alters histone acetylation in the presence of ethylene gas by recruitment of histone modifiers. Activated signalling cascades can alter the epigenetic landscape when signalling factors directly or indirectly communicate with chromatin regulators. For instance, in hypoxic conditions, HIF1α and HIF1β bind to and recruit the histone acetyltransferase p300 and its homologue CREB-binding protein (CBP) to chromatin, resulting in increased local nucleosomal acetylation and transcriptional activation of hypoxia-responsive genes^43^,^44^. Numerous studies have demonstrated that the histone modifiers often reside in multiprotein complexes^45^,^46^,^47^. For instance GCN5 is found in different complexes with different specificities^48^,^49^. From *ENAP1ox* RNA-seq and the phenotype of *ENAP1ox/ein2-5*, it is expected that ENAP1 is also involved in other signalling pathways, potentially depends on its partners. We propose that in response to ethylene, EIN2 CEND is translocated into the nucleus, interacting with ENAP1 and other currently unknown components to guide modifications of specific histone residues. Future studies that identify the proteins complex with the EIN2 CEND and ENAP1 in the presence of ethylene gas will reveal how histone modifiers are recruited to nucleosomes bound to genes regulated by ethylene signalling. In pull-down assays, we found that ENAP1 interacts not only with histone H3, but with histone H2B, indicating that ENAP1 could be involved in other histone modifications, not only limited to histone H3. The identification and characterization of more factors interacting with ENAP1 in different responses will also be of interest.

We provide multiple lines of evidence that EIN2 CEND is required for the regulation of histone acetylation H3K14Ac and H3K23Ac in ethylene signalling. In *ein2-5* mutant seedlings, the basal levels of H3K14Ac and H3K23Ac at subset of ethylene-responsive genes are reduced compared with those in wild-type seedling and no increase in response to ethylene was observed (Fig. 2). *In vitro* immunoprecipitation data indicate that the EIN2 C-terminal end interacts with ENAP1 (Fig. 3). ENAP1, a newly discovered protein, contains a SANT domain that likely interacts with chromatin modifiers. In addition, our data showed that ENAP1 binds to histone H3 (Fig. 4a–c) and that the alteration of histone acetylation caused by ENAP1 overexpression was EIN2 dependent (Fig. 5). Our study also revealed for the first time that an ER membrane protein is required for histone acetylation. Neither the EIN2 C-terminal end nor ENAP1 possess an intrinsic histone acetyltransferase activity domain. Exactly how the EIN2 CEND regulates histone acetylation will require identification of additional EIN2 CEND partners in the nucleus. In addition, to further explore whether and how other histone modifications are involved in ethylene response will also be of interest.

We showed that EIN2 is required for the elevation of histone acetylation at H3K14 and H3K23 in response to ethylene (Figs 2 and 5). Since no EIN3 protein is detectable in the *ein2-5* mutant^24^,^50^, the lack of H3K14 and H3K23 modifications over ethylene-responsive genes in the *ein2-5* and *ENAP1ox/ein2-5* mutants (Figs 2a,b,d–f and 5b) may be due to the absence of EIN2 and transcription factors EIN3 and EIL1. The ChIP-qPCR data has demonstrated that EIN3 and EIL1 affect the elevation of histone acetylation differently than does EIN2 (Figs 2, 5 and 6). One possibility is that the other homologues of EIN3 (EIL1, EIL2, EIL3, EIL4) characterized previously^23^ may be involved in the recruitment of the histone modifiers to specific targets, although they are not functionally identical to EIN3 or EIL1. To test this, acetylation of H3K14 and H3K23 should be examined in the higher order mutants of *EIN3* and its homologues, such as *ein3eil1eil2, ein3eil1eil2eil3* and *ein3eil1eil2eil3eil4*.

Two models have been proposed to explain how the histone modifying activity is selectively targeted to nucleosomes over promoter regions. One hypothesis is that histone modifiers are recruited to particular promoters by sequence-specific DNA-binding proteins. Although a direct interaction between EIN2 CEND and EIN3 was not detected, our data showed that ENAP1 interacts with both EIN2 CEND and EIN3, suggesting that EIN2 CEND, ENAP1 and EIN3 may potentially be part of the same protein complex. EIN3 or another family member could determine the specific targets of histone acetylation. Whereas, we cannot exclude the alternative model that the histone modifiers themselves contain intrinsic preferences for specific histone modifications. For example, the bromodomains in the yeast Swi2/Snf2 remodelers and GCN5 HAT are sufficient to anchor their respective complexes to acetylated promoters in the absence of transcriptional activators^51^. Therefore, it is possible that the EIN2 C-terminal end or the chromatin modifiers recruited by the EIN2 CEND have intrinsic preferences for specific targets. Further comprehensive studies will be critical to uncover the mechanistic details that govern hormone signalling properties in plants.

## Methods

### Plant growth conditions and hypocotyl measurements

*Arabidopsis* seeds were surface-sterilized in 50% bleach with 0.01% Triton X-100 for 15 min and washed five times with sterile, doubly distilled H_2_O before plating on MS medium (4.3 g MS salt, 10 g sucrose, pH 5.7, 8 g phytoagar per liter) with or without addition of 10 μM 1-aminocyclopropane-1-carboxylic acid (ACC, Sigma), the biosynthetic precursor to ethylene. After 3–4 days of cold (4 °C) treatment, the plates were wrapped in foil and kept in at 24 °C in an incubator before the phenotypes of seedlings were analyzed. For propagation, seedlings were transferred from plates to soil (Pro-mix-HP) and grown to maturity at 22 °C under 16-h light/8-h dark cycles. Ethylene treatment of *Arabidopsis* seedlings was performed by growth of seedlings on MS plates in air-tight containers in the dark supplied with either a flow of hydrocarbon-free air (Zero grade air, AirGas) or 4 h hydrocarbon-free air with 10 p.p.m.) ethylene as previously described^12^. For hypocotyl length measurements, 3-day-old seedlings were scanned using an Epson Perfection V700 Photo scanner, and hypocotyls were measured using NIH Image (http://rsb.info.nih.gov/nih-image/).

### Ethylene inhibitor assay

*Arabidopsis* seeds were surface-sterilized in 50% bleach with 0.01% Triton X-100 for 15 min and washed five times with sterile, doubly distilled H_2_O before plating on MS medium (4.3 g MS salt, 10 g sucrose, pH 5.7, 8 g phytoagar per litre) with or without addition of 10 μM Aminoethoxyvinylglycine (AVG, Sigma), the inhibitor ethylene. Then the plates were taken into the air-tight container in the dark at 22 °C and supplied with a flow of hydrocarbon-free air (Zero grade air, AirGas) for 3 days. Total RNA was extracted using a RNeasy Plant Kit (Qiagen) from 3 days etiolated seedlings. First-strand cDNA was synthesized using Superscript III First-Strand cDNA Synthesis Kit (Invitrogen). Realtime PCR was performed with the LightCycler 480 SYBR Green I Master (Roche) following the manufacturer's instructions. PCR reactions were performed in triplicate on a Roche 96 Thermal cycler. The expression level was normalized to that of a *UBQ10* control.

### Histone extraction and western blot

Histone extraction was performed as described previously^52^. Briefly seedlings were ground to powder in a mortar cooled with liquid nitrogen, and the powder was suspended in NIB buffer (250 mM sucrose, 60 mM KCl, 15 mM NaCl, 5 mM MgCl_2_, 1 mM CaCl_2_,15 mM Pipes, pH 6.8, 0.8% Triton X-100). The suspension was centrifuged (13,000*g*, 10 mins), and the pellet was resuspended in 0.4 M H_2_SO_4_ and incubated for at least 2 h at 4 °C. The preparation was centrifuged again before acetone was added to precipitate histone proteins. The preparation was kept at −20 °C overnight, and then proteins were dissolved in 4 M urea, separated on SDS–PAGE, and analyzed by western blotting. The antibodies used in the western blotting were anti-H3K9Ac (Millipore; 07-352, 1:3,000 dilution), anti-H3K14Ac (Millipore; 07-353, 1:2,000 dilution), anti-H3K18Ac (Millipore; 07-354, 1:2,500 dilution), anti-H3K23Ac (Millipore; 07-355, 1:3,000 dilution), anti-H3K27Ac (Millipore; 07-360, 1:3,000 dilution), anti-H3Ac (Millipore; 06-599, 1:3,000 dilution), and anti-H4Ac (Millipore; 06-598, 1:3,000 dilution). For histone peptide dot blot: Commercial synthesized histone peptides (all the histone peptides were custom synthesized by Active Motif, 5 μg per peptide were used) as indicated in figures were spotted on nitrocellulose membrane, and the membrane was incubated with PBS buffer which contain *in vitro* purified GST-ENAP1 protein for 1 h in 4° cold room, then subjected to anti GST antibody (Cell Signaling 91G1, 1:5,000) for immunoblot.

### Plant protein extraction

*Arabidopsis* seedlings were harvested and immediately frozen in liquid N_2_ and stored at −80 °C until processing. For total plant protein extraction, frozen seedlings were ground in liquid N_2_ and mixed with extraction buffer (100 mM Tris–HCl, pH 7.5, 100 mM NaCl, 5 mM EDTA, 10 mM *N*-ethylmaleimide, 5 mM DTT, 10 mM β-mercaptoethanol, 1% SDS and protease inhibitors from Sigma P8465), and centrifuged at 10,000*g* for 10 min at 4 °C. The supernatant was collected for further analysis.

### Gene expression experiments

RNA from wild-type, *ctr-1-1*, and *EIN2*^*S645A*^*-YFP-HA* transgenic lines treated with 4 h ethylene gas or hydrocarbon-free air were isolated following the manufacturer's recommendation using the RNeasy Plant Kit (Qiagen). cDNA sequencing libraries were prepared according to the instructions included in the Illumina TruSeq v2 library preparation kit. Reads were mapped using TopHat, and analyzed using Cufflinks^53^. Differentially expressed genes were identified by fragments per kilobase per million reads (FPKM) filter<0.1, requiring a twofold change comparing the indicated conditions with P≤0.05 after Benjamini–Hochberg correction.

### ChIP-seq

Chromatin immunoprecipitation was performed according to a published protocol^25^. Briefly 3-day-old etiolated seedlings treated with air or 4 h ethylene were harvested and crosslinked in 1% formaldehyde, and the chromatin was isolated. The indicated antibodies [anti-H3K14Ac (Millipore; 07-353), anti-H3K23Ac (Millipore; 07-355), anti-H3Ac (Millipore; 06-599) and anti-H4Ac (Millipore; 06-598)] together with Magnetic Protein G Beads (Promega, G747A) were added to the sonicated chromatin followed by incubation overnight to precipitate bound DNA fragments (2 μg antibody per each chip reaction for all the antibodies used in this paper). DNA was eluted and amplified by primers corresponding to genes of EIN3-R and EIN3-NR. Primers used in the paper are listed in Supplementary Table 1.

### ChIP-seq data analysis

Chromatin-immunoprecipitated DNA was sequenced using an Illumina HiSeq 2000 platform according to standard operating procedures. Single-end 51-bp reads were first mapped to the *Arabidopsis* genome (TAIR10)^54^ using Bowtie2 software (version 2.1.0)^55^ with default parameters. The quality control of Chip-seq was shown in Supplementary Fig. 7. Duplicate reads were removed using SAMtools^56^. For each histone modification in each condition, mapped reads were pooled across ChIP-seq replicates as described^57^. Pooled reads were normalized as reads per kilobase per million mapped reads (RPKM) in windows of 50 bp using deep Tools^58^, and then were visualized with the Integrative Genomics Viewer (IGV)^59^ (Supplementary Table 3). In addition to validate our peak calling result by pooled peaks, we also did analysis using overlapped differential peaks, and the result is very similar to that called by using pooled reads. On the basis of a recent publication^57^, we used pooled reads results in this paper. Peaks significantly enriched in ChIP-seq tags were identified by Model-based Analysis for ChIP-Seq (MACS2, version 2.1.0.20150603; parameters: --nomodel, -p 0.01)^60^. Differential peaks were identified using ‘MAnorm' method^61^. For this method, the normalized *M* value (*M*=log2 (read density in C_2_H_4_ treated sample per read density in air treated sample) represents log2-transformed fold changes of enrichment intensities at each peak region^61^,^62^,^63^. Thus, an absolute threshold value of M≥0.4 was used to select differentially enriched peaks. Genes within 2 kb of the peak regions were marked as associated genes. Biological functions of associated genes were assessed by agriGO^64^.

### Western blotting analysis

Proteins were resolved by SDS–PAGE and electroblotted onto a nitrocellulose membrane and probed with the indicated primary antibodies and then with secondary goat anti-rabbit (Bio-Rad 170-6515) or goat anti-mouse (Bio-Rad 170-6516) antibodies conjugated with horseradish peroxidase. The signals were detected by a chemiluminescence reaction using the SuperSignal kit (Pierce). Polyclonal anti-EIN2 antibodies were used at dilution of 1:4,000. Polyclonal anti-histone H3 (BioMol) was used at dilution of 1:5,000. Monoclonal anti-HA (Cell Signaling) was used at dilution of 1:5,000.

### Gene expression analysis

Total RNA was extracted using a Qiagen Plant Total RNA Kit (Sigma) from 3-day-etiolated seedlings treated with air or 4 h ethylene gas. First-strand cDNA was synthesized using Invitrogen Superscript III First-Strand cDNA Synthesis Kit. cDNAs were combined with SYBR master mix from BIOLINE for PCR. PCR reactions were performed in triplicate on a Roche 96 Thermal cycler. Primers used in the paper are listed in Supplementary Table 1.

### Pull-down

GST fusion proteins (GST-CD3 and the negative control GST protein) purified using glutathione Sepharose 4B (GE Healthcare) were washed using pull-down buffer (50 mM Tris–Cl, pH 8, 150 mM NaCl, 0.5 mM EDTA, 0.5% Tween 200, and protease inhibitor cocktail). Histones from calf thymus (Sigma, H9250) were added and incubated for 1 h at 4 °C. After washing five times with pull-down buffer, precipitated Sepharose beads were collected by brief centrifugation (2,000*g*, 2 min) and then resuspended in protein extraction buffer. Proteins were separated by SDS–PAGE and detected with the H3 antibody.

### Co-immunoprecipitation assay

Three-day-old etiolated seedlings (*ENAP1-YFP-HA*; *EIN3-Flag*/*ENAP1-YFP-HA*) treated with or without ethylene gas were harvested. Proteins were extracted with co-IP buffer (50 mM Tris–Cl, pH 8, 150 mM NaCl, 1 mM EDTA, 0.1% Triton X-100, and protease inhibitor cocktail). After incubation on ice for 30 min, plant extracts were centrifuged. Cleared extract was combined with anti-EIN2 antibody or anti-Flag antibody overnight at 4 °C, then Magnetic Protein G Beads (Promega, G747A) were added, and samples were incubated for 1.5 h at 4 °C. After washing five times with co-IP buffer, magnetic beads were collected and then resuspended in protein extraction buffer. Proteins were separated by SDS–PAGE and detected with the EIN2 antibody to detect CD3 interaction with EIN2 or with HA antibody to detect the interaction between ENAP1 and EIN2 CEND or EIN3.

Full uncropped versions of all gel/blot are in additional Supplementary Fig. 8

### Data availability

ChIP-seq data presented in this manuscript have been deposited in the NCBI GEO database under accession code GSE77396. The authors declare that all other data supporting the findings of this study are available within the article and its Supplementary information files. IGV files and any other supporting data are available from the corresponding author upon request.

## Additional information

**How to cite this article**: Zhang, F. *et al.* EIN2-dependent regulation of acetylation of histone H3K14 and non-canonical histone H3K23 in ethylene signalling. *Nat. Commun.* **7**, 13018 doi: 10.1038/ncomms13018 (2016).

## Supplementary Material

Supplementary Information Supplementary Figures 1 – 8 and Supplementary Tables 1 - 3

Supplementary Data 1 Peak files of H3K9Ac, H3K14Ac and H3K23Ac in Col, ein2-5 treated with air or ethylene

Supplementary Data 2 Ethylene induced diff-Peak (H3K14c and H3K23Ac) associated genes and GO analysis in Col

Supplementary Data 3 Ethylene induced diff-Peak (H3K14c and H3K23Ac) associated genes and GO analysis in ein2-5

Supplementary Data 4 ENAP1ox altered gene list and GO analysis.
