# Supplementary material for: EIN2-dependent regulation of acetylation of histone H3K14 and non-canonical histone H3K23 in ethylene signalling
Source: Nat Commun. 2016 Oct 3;7:13018. doi: 10.1038/ncomms13018 (PMC5063967; doi:10.1038/ncomms13018)
Supplement: Supplementary Information — Supplementary Figures 1 – 8 and Supplementary Tables 1 - 3 [file ncomms13018-s1.pdf]

Supplementary information

Supplementary Figures and Figure legends

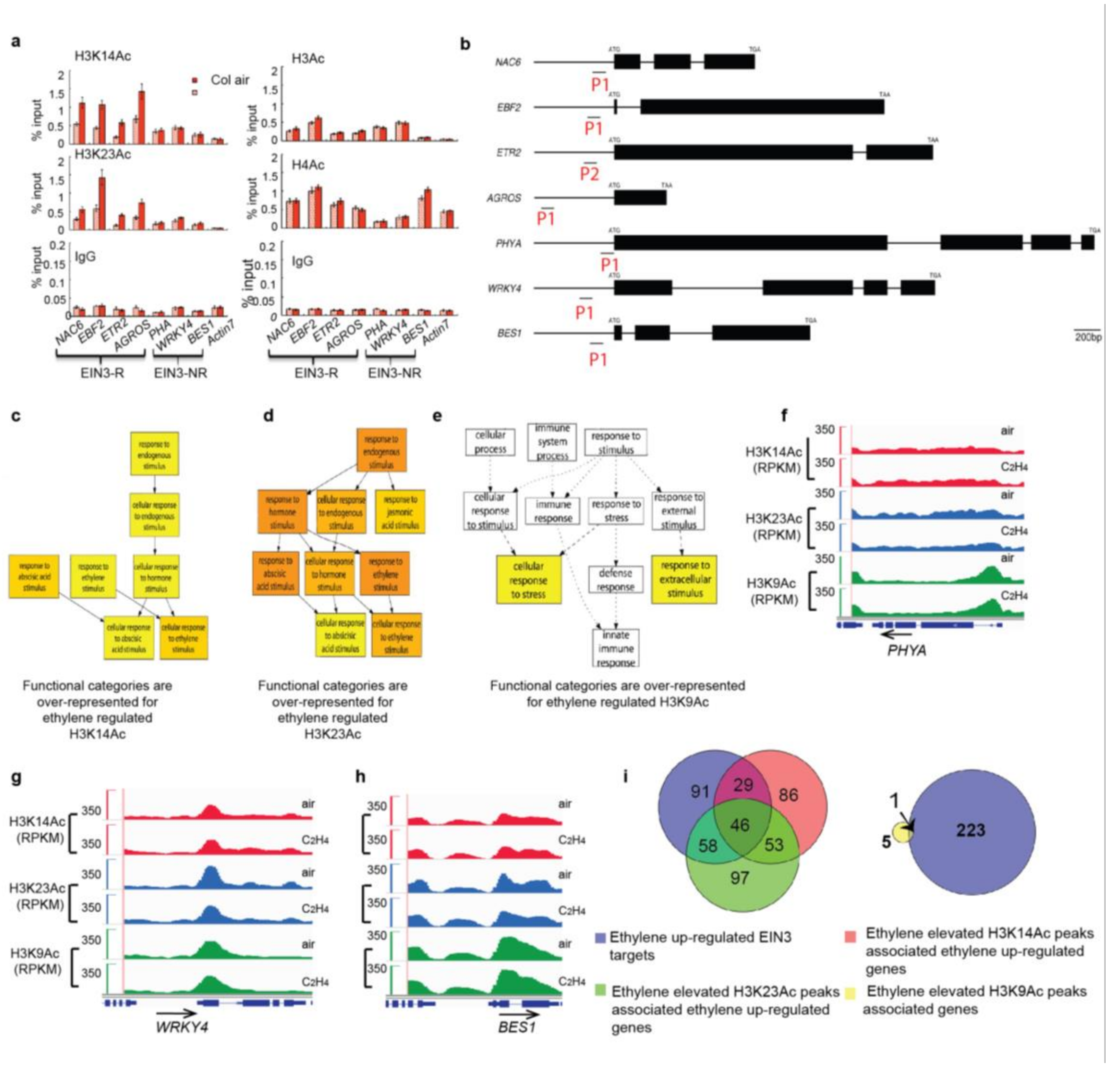

Supplementary figure 1. (a) ChIP quantitative real time PCR detection of H3Ac, H4Ac, H3K14Ac and H3K23Ac enrichment in Col-0 treated with air or ethylene gas. Col wild-type seedlings were grown in dark for 3 d with or without ethylene treatment. Precipitation with IgG preimmune serum served as a control. Data represent the relative to input. Each experiment has three biology replicates with similar result. (b). Diagrams indicate the localization of the primers used for ChIP-PCR. (c-e) Gene Ontology (GO) analyses of ethylene regulate H3K14Ac and H3K19Ac associated genes showing the enrichment of response to ethylene stimulus. (f-h) Standard ChIP-seq assays showing

that the elevated enrichment of H3K14Ac and H3K23Ac in EIN3-NR genes in Col-0 treated with ethylene and no significant enrichment of H3K9Ac is observed. Binding levels are indicated by reads per kilobase per million reads in sample (RPKM). (i) Overlapping between ethylene up-regulated EIN3 targets and ethylene elevated H3K14Ac or H3K23Ac associated ethylene-regulated genes, showing that H3K14Ac or H3K23Ac in ethylene regulated EIN3 targets are specifically induced by ethylene. (Ethylene up regulated EIN3 targets is the overlapped genes of EIN3-R from elife Chang et al., and ethylene up regulated gene from Qiao et al., 2012). EIN3-R represents ethylene regulated EIN3 targets. EIN3-NR represents non-ethylene regulated EIN3 targets.

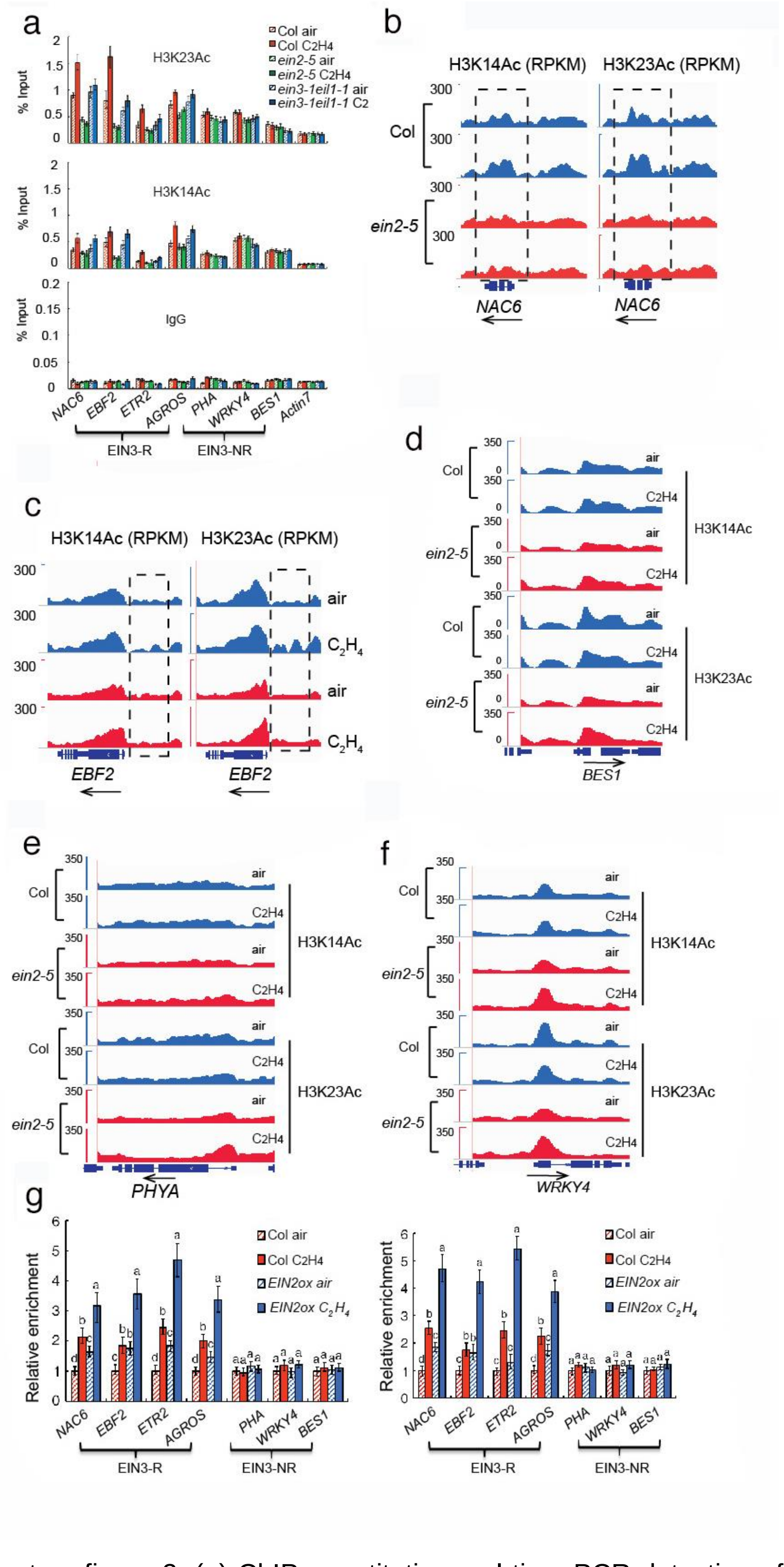

Supplementary figure 2. (a) ChIP quantitative real time PCR detection of H3K14Ac and

H3K23Ac enrichment in Col-0, *ein2-5, ein3-1eil1-1* treated with air or ethylene gas. Precipitation with IgG preimmune serum served as a control. Data represent the ChIP enrichment relative to input. Each experiment has three biology replicates with similar result. (b-f) Standard ChIP-seq assays showing that the elevated enrichment of H3K14Ac and H3K23Ac in T-R and T-NR genes in Col-0 treated with ethylene. Binding levels are indicated by reads per kilobase per million reads in sample (RPKM). Col-0 seedlings grown in the dark for 3 days with or without ethylene treatment for ChIP-seq. (g). ChIP quantitative real time PCR detection of H3K14Ac and H3K23Ac enrichment in Col-0, *ENAP1ox* treated with air or ethylene gas. Precipitation with IgG preimmune serum served as a control. Data represent the ChIP enrichment relative to input. Each experiment has three biology replicates with similar result.

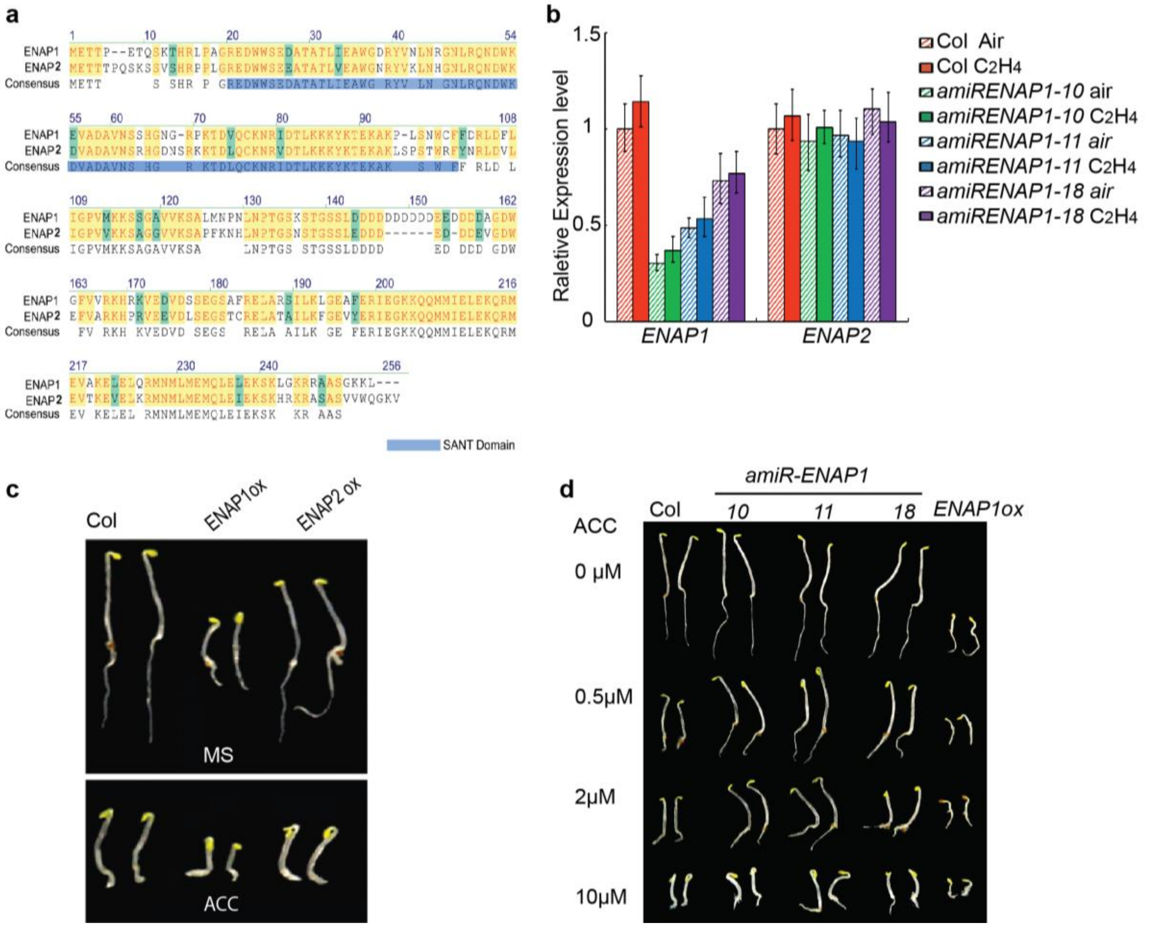

Supplementary figure 3. (a) Alignment of ENAP1 and ENAP2 at amino acid level showing the similarity of the two proteins. (b) Quantitative PCR assay shows artificial microRNA knock down gene expression of ENAP1, not ENAP2. Total RNAs were extracted from 3-day old etiolated seedlings of the plants indicated in the figure. (c-d) Ethylene response phenotype of *ENAP1* gain function (*ENAP1ox*) and independent knocking-down mutants of *amiR-ENAP1*. Ethylene response phenotype of 3-d-old etiolated seedlings of *ENAP1ox, amiR-ENAP1* plants were grown on MS with or without 10μM ACC before photographed.

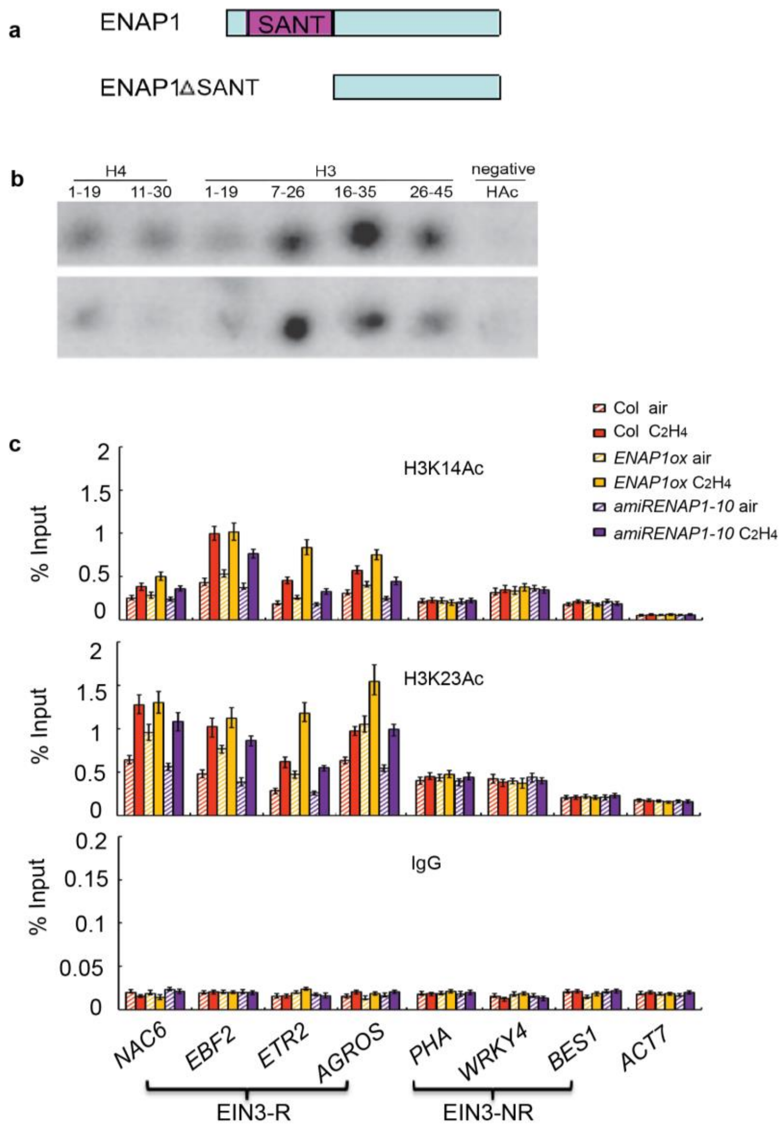

Supplementary figure 4. ENAP1 interacts with Histone H3 and regulate histone acetylation of H3K14 and H3K23. (a) Diagram showing the construct for expression the fully length of ENAP1 and the truncated ENAP1 without SANT domain (ENAP1ΔSANT). (b) Dot blot detecting ENAP1 with different histone peptides. Commercial histone peptides were spotted on nitro cellular membrane and the blotting was done using purified ENAP1 as probe. (c) ChIP quantitative real time PCR detection of H3K14Ac and H3K23Ac enrichment in Col-0, *ENAP1ox and amiRENAP1* treated with air or ethylene gas. Precipitation with IgG preimmune serum served as a control. Data represent ChIP enrichment relative to input. Each experiment has three biology replicates with similar result.

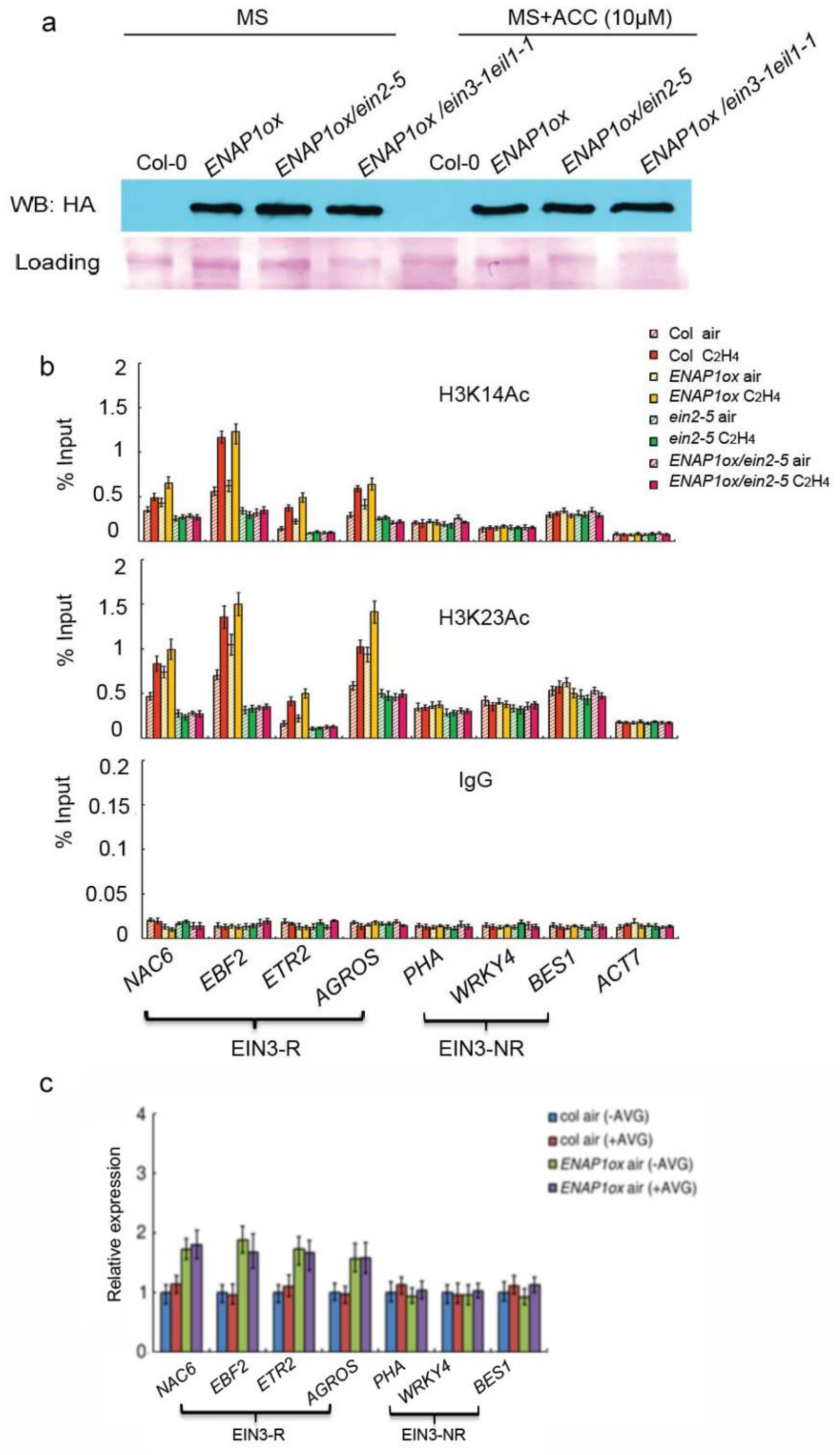

Supplementary figure 5. ENAP1 plays its roles in ethylene in an EIN2 dependent manner. (a) Western blot of ENAP1 showing the protein level of ENAP1 is not altered by ethylene, EIN2 or EIN3. The total protein extractions from 3-day old etiolated seedlings of the plants indicated in the figure subjected to immunoblotting with anti-HA antibody. (b) ChIP quantitative real time PCR detection of H3K14Ac and H3K23Ac enrichment in Col-0,

ENAP1ox and ENAP1ox/ein2-5 treated with air or ethylene gas. Precipitation with IgG preimmune serum served as a control. Data represent the ChIP enrichment relative to input. Each experiment has three biology replicates with similar result. (c) quantitative real time PCR detection of gene expression in Col-0 and *ENAP1ox* grown on MS with or without AVG, showing that ethylene biosynthesis is not influenced in *ENAP1ox.*

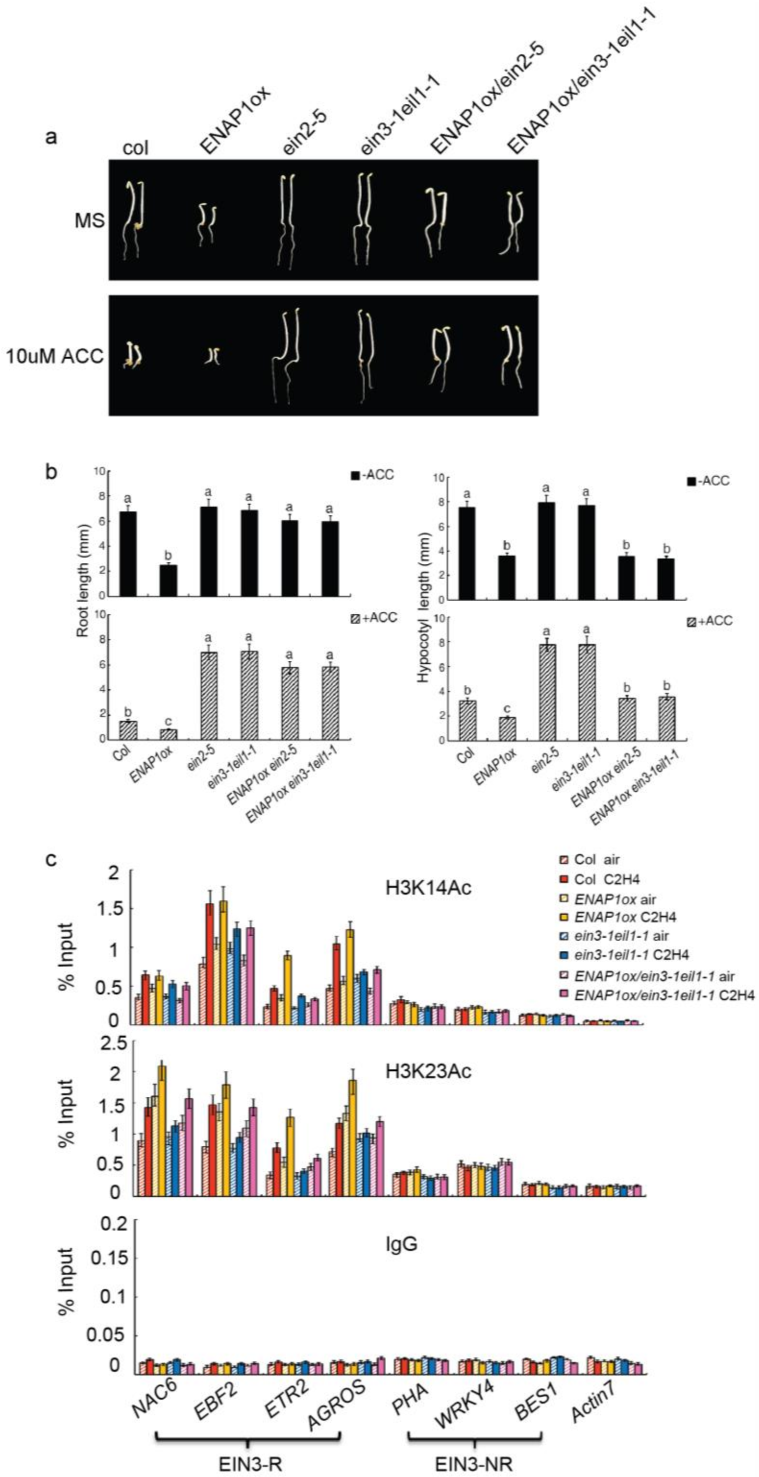

Supplementary figure 6. EIN3 is required for the function of ENAP1 (a) The ethylene response phenotype of *ENAP1ox* in *ein3-1eil1-1* mutant. The 3-day old etiolated seedlings of the plants indicated in the figure were grown on MS with (right panel) or without (left panel) 10μM ACC before photographed. (b) Measurement of the roots,

hypocotyls of 3-day old seedlings of the plants indicated grown on MS with or without 10μM ACC. (c) ChIP quantitative real time PCR detection of H3K14Ac and H3K23Ac enrichment in Col-0, *ENAP1ox and ENAP1ox/ein3-1eil1-1* treated with air or ethylene gas. Precipitation with IgG preimmune serum served as a control. Data represent the ChIP enrichment relative to input. Each experiment has three biology replicates with similar result.

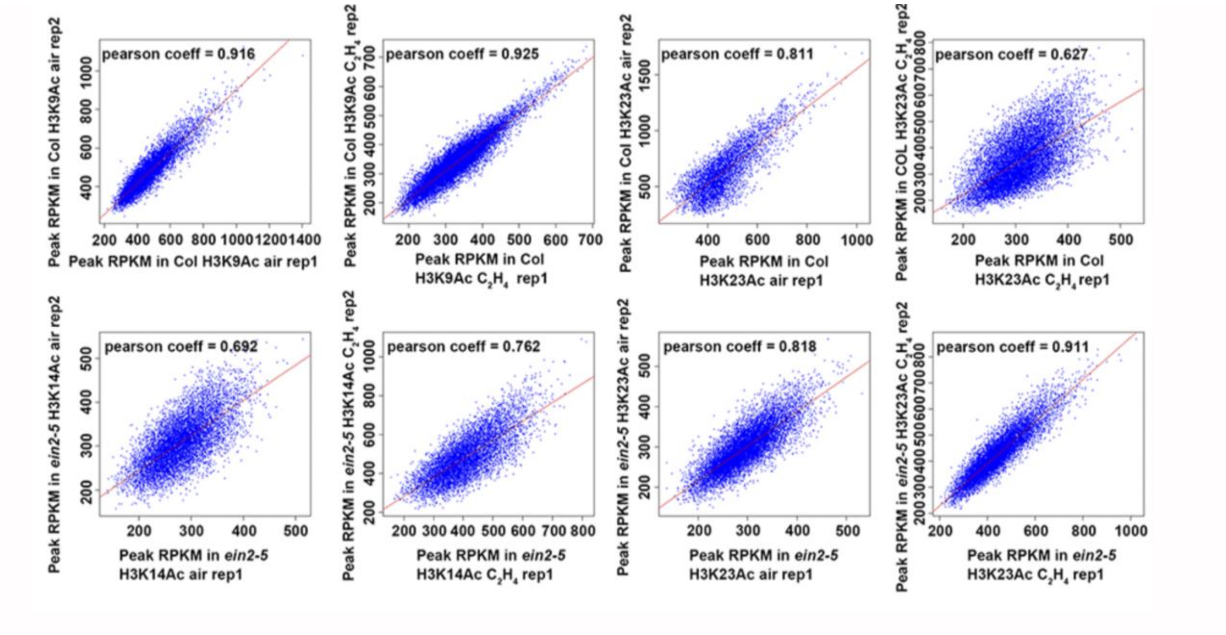

Supplementary figure 7. Pearson correlation of two biological replications of peaks as indicated in the figure. For each region that enriched histone modifications, the RPKMs for each sample were calculated and R scripts were used to analyze the correlation between samples.

Supplementary Figure 8. Original western blots, the black frames indicating the portion showed in main figures.

Figure 1a

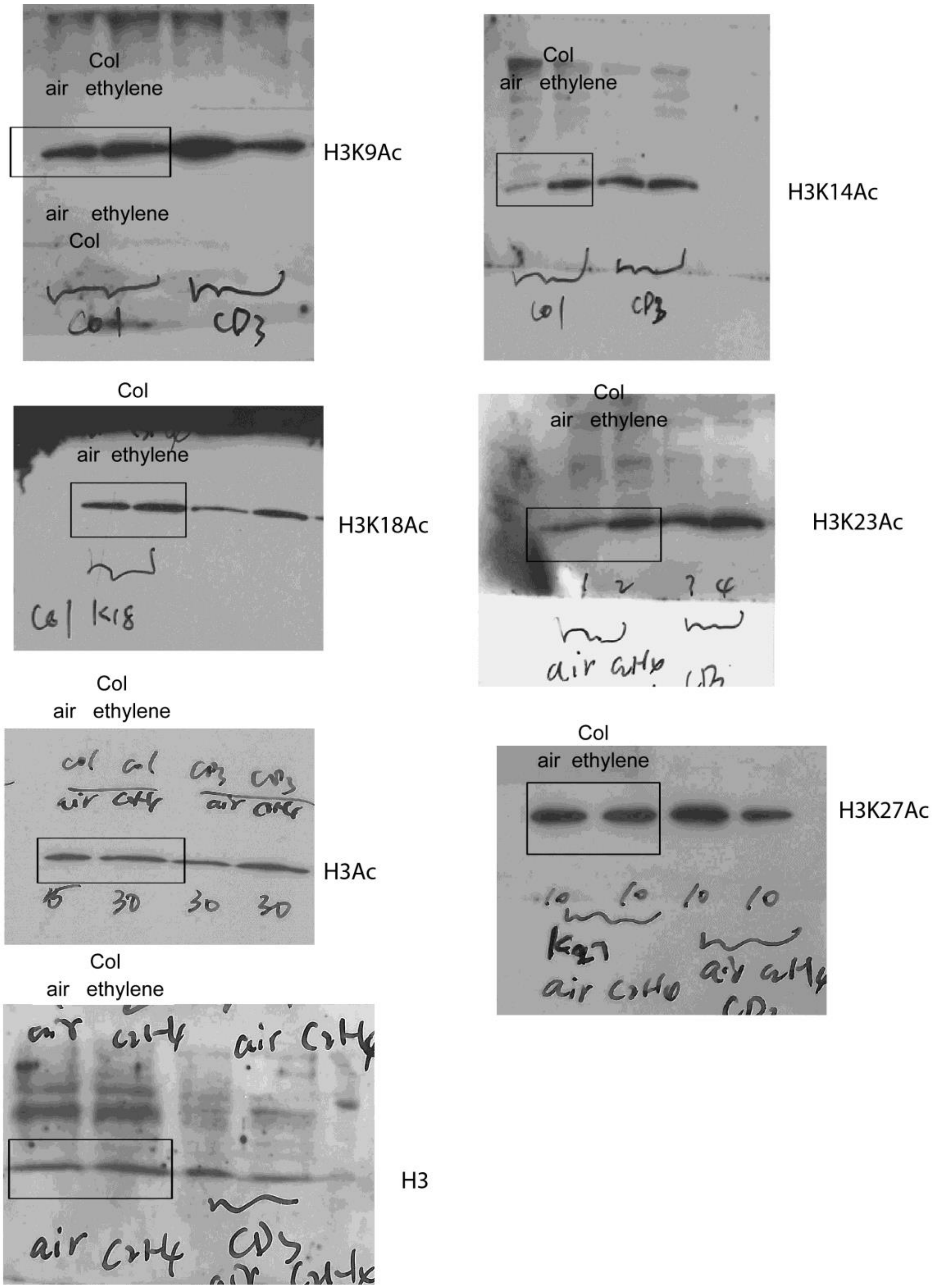

Figure 3B

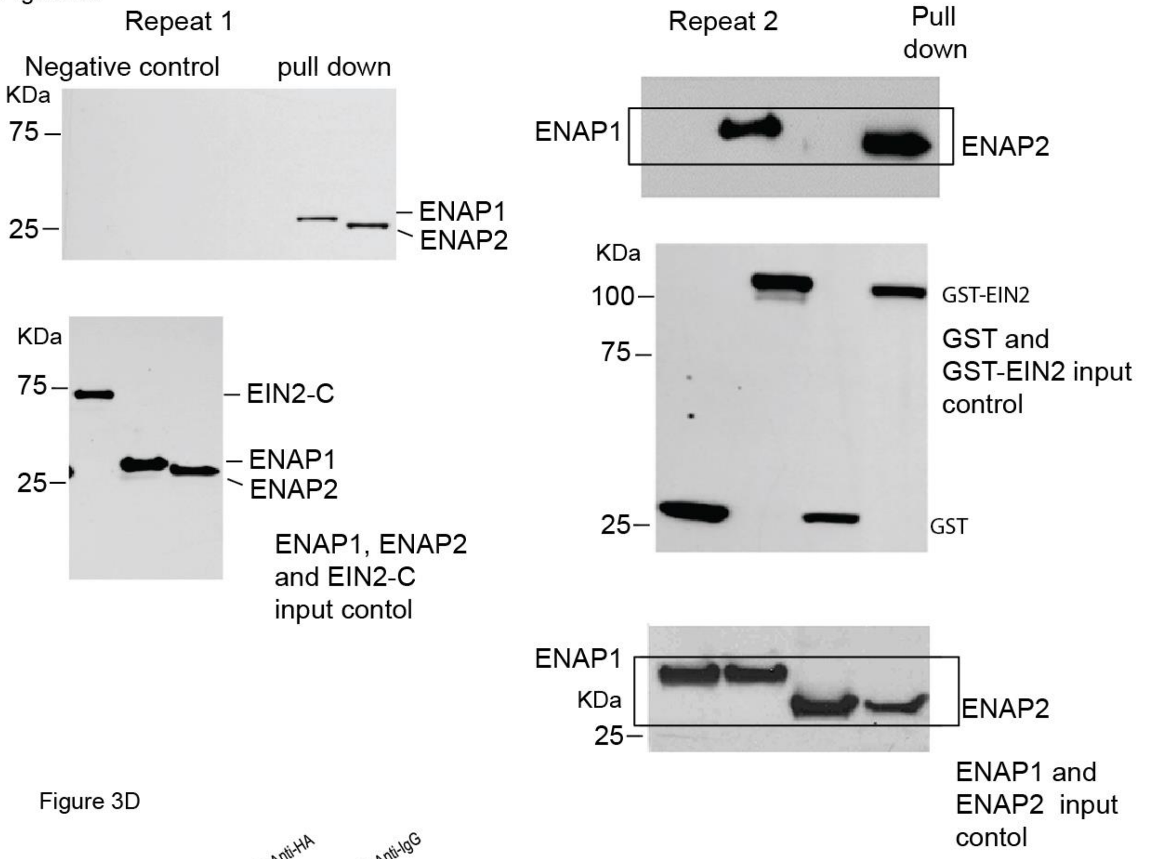

Figure 3D

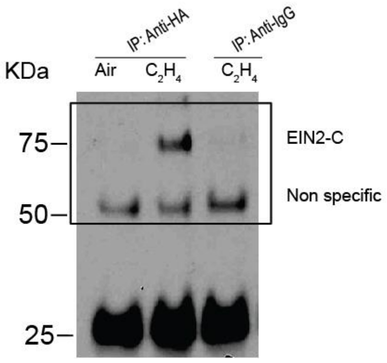

Figure 4A

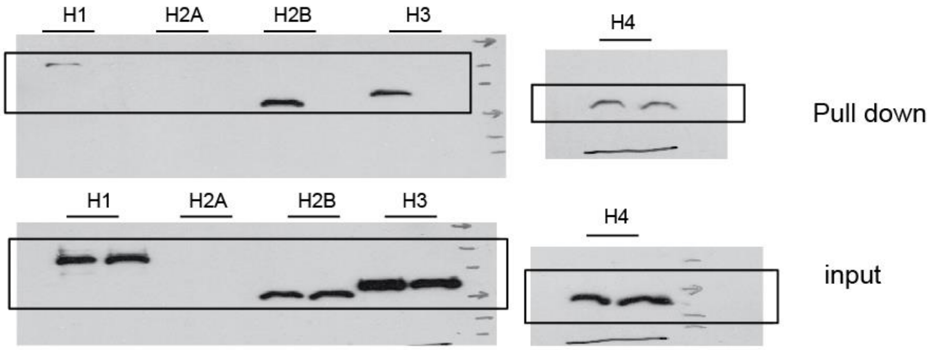

Figure 4C

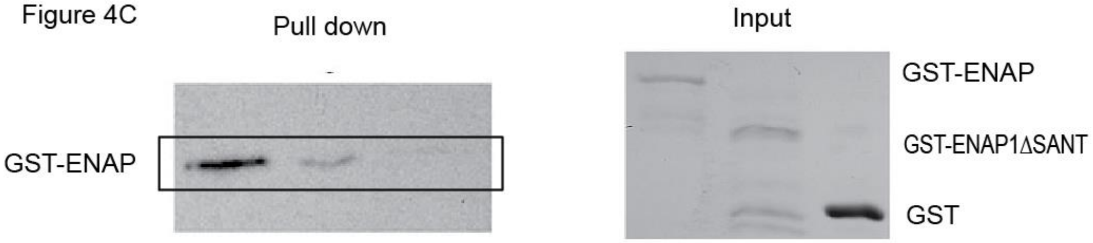

Figure 4E

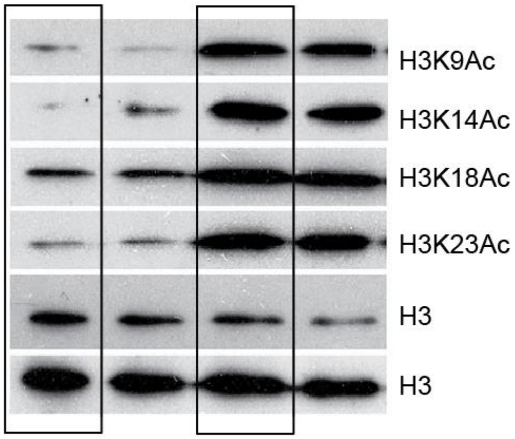

Figure 6B

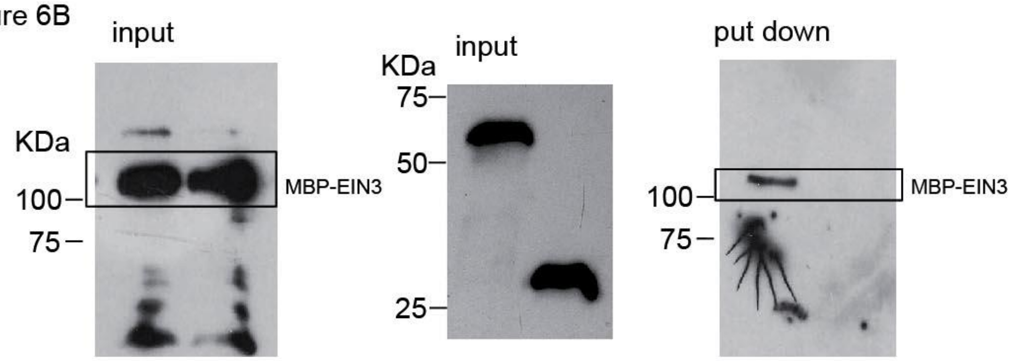

Figure 6C

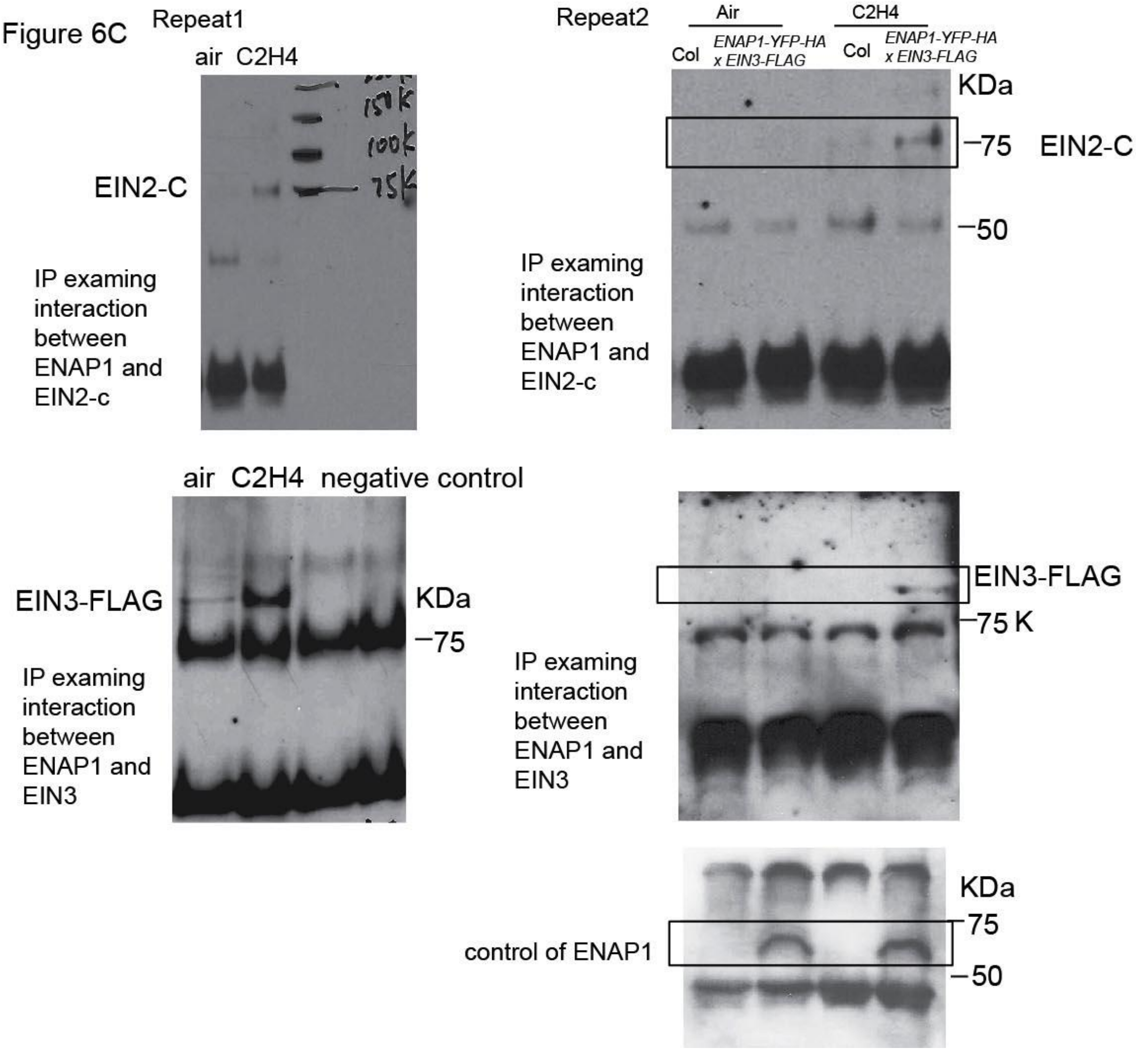

Supplementary Table 1: Primers for qPCR and ChIP-PCR

Primer Name	Sequence	Purpose
NAC6 ChIP-qPCR F	TAGAGAGGAGCTTCGTTGCTC	ChIP-qPCR
NAC6 ChIP-qPCRR	GTTTGGAGACGAAGAGGGAAG	ChIP-qPCR
EBF2 ChIP-qPCR F	AACTTCCACCGTACACCTCTC	ChIP-qPCR
EBF2 ChIP-qPCRR	GAACCCTAGAAGATTAGGGC	ChIP-qPCR
ETR2 ChIP-qPCR F	CTTCTATGAGAGGAAGATCGGAATG	ChIP-qPCR
ETR2 ChIP-qPCR R	GATCTCTCATCGACCTGTGAAAT	ChIP-qPCR
AGROS ChIP-qPCR F	CCTTGTGATTGTGGAGGAAGA	ChIP-qPCR
AGROS ChIP-qPCR R	GGCTGACAAATGTGAGGAGA	ChIP-qPCR
PHYA ChIP-qPCR F	GCATTCGCTTGTATGACTGGTG	ChIP-qPCR
PHYA ChIP-qPCR R	TCCTGACACAGAGACAAGAC	ChIP-qPCR
WRKY4 ChIP-qPCR F	CCAGATCCGTTTTAAGACCAG	ChIP-qPCR
WRKY4 ChIP-qPCR R	TGAATCCGTGTCGTGTTTCG	ChIP-qPCR
BES1 ChIP-qPCR F	CAGCTCCTTTTCTTACACAG	ChIP-qPCR
BES1 ChIP-qPCR R	CGTGTCGATGATGAATGGTG	ChIP-qPCR
Actin7 ChIP-qPCR F	CGGGCTAATTCATTTGAACC	ChIP-qPCR
Actin7 ChIP-qPCR R	GGTGACCTGGCTTTCACACT	ChIP-qPCR
NAC6 qF	CTCTGTTTTACTCGGATCCTC	qPCR
NAC6 qR	ACGAATCACGACCGTCGAAAG	qPCR
EBF2 qF	TGATGTTGGTCTTGGTGCTGTTGC	qPCR
EBF2 qR	ATTCCAGGACACCGTGAAAGGTCA	qPCR
ETR2 qPCR F	GGAGTTGTGAGAAAGCCAGTGG	qPCR
ETR2 qPCR R	GAGAAGTTGGTCAGCTTGC	qPCR
AGROS qPCR F	TGGTCTAACGGCATCTCTG	qPCR
AGROS qPCR R	GAGAAGAAGGCATGAAGGC	qPCR
PHYA qPCR F	GTTAGCCGGAAACTGGTGAAG	qPCR
PHYA qPCR R	CTACTTGTTTGCTGCAGCG	qPCR
WRKY4 qPCR F	GCTAAATCAAGCAGCCATGC	qPCR
WRKY4 qPCR R	AACGGGCTGTTGCTGCTGCTGT	qPCR
BES1 qPCR F	GCAGCAATCCAAGAGATTGG	qPCR
BES1 qPCR R	CAAGCGTGAGCTCTAGATC	qPCR
UBQ1 qPCR F	TTCCTTGATGATGCTTGCTC	qPCR
UBQ1 qPCR R	TTGACAGCTCTTGGGTGAAG	qPCR

Supplementary Table 2: Summary of reads and peaks in different samples.

				Diff_peaks without cutoff (C_2H_4/air)	
Sample_ID	Total number of reads	Unique mapped reads	% unique mapped reads	Up-regulated	Down-regulated
Col_C_2H_4_IgG	43265142	20840723	48.17		
Col_air_IgG	55081643	23179111	42.08		
Col_H3K9Ac_ C_2H_4_rep1	22172156	18651798	84.12	15163	13619
Col_H3K9Ac_ C_2H_4_rep2	20601350	17432693	84.62		
Col_H3K9Ac_ air_rep1	19953603	16633710	83.36		
Col_H3K9Ac_ air_rep2	22809256	19536260	85.65		
Col_H3K14Ac _C_2H_4	41228515	24543770	59.53	12714	5614
Col_H3K14Ac_ air	22781109	18289996	80.29		
Col_H3K23Ac_ C_2H_4_rep1	36274865	28974579	79.88	11081	6881
Col_H3K23Ac_ C_2H_4_rep2	19454562	15135595	77.8		
Col_H3K23Ac_ air_rep1	30136791	24242184	80.44		
Col_H3K23Ac_ air_rep2	17247705	13508694	78.32		
*ein2-5*_C_2H_4_IgG	17931071	8204864	45.76		
*ein2-5*_air_IgG	41350476	26216724	63.4		
*ein2-5*_H3K14Ac _C_2H_4_rep1	25928061	22649514	87.36	7706	9589
*ein2-5*_H3K14Ac _C_2H_4_rep2	29112612	25310920	86.94		
*ein2-5*_H3K14Ac _air_rep1	20978999	16797682	80.07		
*ein2-5*_H3K14Ac _air_rep2	19318232	15017654	77.74		
*ein2-5*_H3K23Ac _C_2H_4_rep1	18607436	16015424	86.07	10680	12441
*ein2-5*_H3K23Ac_ C_2H_4_rep2	26857786	23570931	87.76		
*ein2-5*_H3K23Ac_ air_rep1	27366957	22222486	81.2		
*ein2-5*_H3K23Ac_ air_rep2	25866283	20272199	78.37		

Supplementary Table 3: Quality control of ChIP-seq:

	Col_H3K9Ac		Col_H3K23Ac		*ein2-5*_H3K14Ac		*ein2-5*_H3K23Ac	
	air	C2H4	air	C2H4	air	C2H4	air	C2H4
rep1_total_peaks	11641	18034	5347	13946	10326	8540	14520	13076
rep2_total_peaks	11928	18256	9817	17074	15106	12043	14759	12150
rep1_overlapped_peakswith rep2	10908	17321	4559	11562	7986	7551	11768	11213
percentage of overlap	0.94	0.96	0.85	0.83	0.773	0.883	0.813	0.86
